# Antifungal plant flavonoids identified *in silico* with potential to control rice blast disease caused by *Magnaporthe oryzae*

**DOI:** 10.1371/journal.pone.0301519

**Published:** 2024-04-05

**Authors:** Abu Tayab Moin, Tanjin Barketullah Robin, Rajesh B. Patil, Nurul Amin Rani, Anindita Ash Prome, Tahsin Islam Sakif, Mohabbat Hossain, Dil Umme Salma Chowdhury, Shah Samiur Rashid, A. K. M. Moniruzzaman Mollah, Saiful Islam, Mohammad Helal Uddin, Mohammad Khalequzzaman, Tofazzal Islam, Nazneen Naher Islam

**Affiliations:** 1 Department of Genetic Engineering and Biotechnology, Faculty of Biological Sciences, University of Chittagong, Chattogram, Bangladesh; 2 Faculty of Biotechnology and Genetic Engineering, Sylhet Agricultural University, Sylhet, Bangladesh; 3 Department of Pharmaceutical Chemistry, Sinhgad Technical Education Society’s, Sinhgad College of Pharmacy, Pune, Maharashtra, India; 4 Department of Computer Science and Electrical Engineering, West Virginia University, Morgantown, WV, United States of America; 5 Department of Biochemistry and Biotechnology, University of Science and Technology Chittagong (USTC), Chattogram, Bangladesh; 6 Department of Biological Sciences, Asian University for Women (AUW), Chattogram, Bangladesh; 7 Chattogram Laboratories, Bangladesh Council of Scientific and Industrial Research (BCSIR), Chattogram, Bangladesh; 8 Department of Applied Chemistry and Chemical Engineering, University of Chittagong, Chittagong, Bangladesh; 9 Bangladesh Rice Research Institute, Gazipur, Bangladesh; 10 Institute of Biotechnology and Genetic Engineering (IBGE), Bangabandhu Sheikh Mujibur Rahman Agricultural University (BSMRAU), Gazipur, Bangladesh; CPRI: Central Potato Research Institute, INDIA

## Abstract

Rice blast disease, caused by the fungus *Magnaporthe oryzae*, poses a severe threat to rice production, particularly in Asia where rice is a staple food. Concerns over fungicide resistance and environmental impact have sparked interest in exploring natural fungicides as potential alternatives. This study aimed to identify highly potent natural fungicides against *M*. *oryzae* to combat rice blast disease, using advanced molecular dynamics techniques. Four key proteins (CATALASE PEROXIDASES 2, HYBRID PKS-NRPS SYNTHETASE TAS1, MANGANESE LIPOXYGENASE, and PRE-MRNA-SPLICING FACTOR CEF1) involved in *M*. *oryzae*’s infection process were identified. A list of 30 plant metabolites with documented antifungal properties was compiled for evaluation as potential fungicides. Molecular docking studies revealed that 2-Coumaroylquinic acid, Myricetin, Rosmarinic Acid, and Quercetin exhibited superior binding affinities compared to reference fungicides (Azoxystrobin and Tricyclazole). High throughput molecular dynamics simulations were performed, analyzing parameters like RMSD, RMSF, Rg, SASA, hydrogen bonds, contact analysis, Gibbs free energy, and cluster analysis. The results revealed stable interactions between the selected metabolites and the target proteins, involving important hydrogen bonds and contacts. The SwissADME server analysis indicated that the metabolites possess fungicide properties, making them effective and safe fungicides with low toxicity to the environment and living beings. Additionally, bioactivity assays confirmed their biological activity as nuclear receptor ligands and enzyme inhibitors. Overall, this study offers valuable insights into potential natural fungicides for combating rice blast disease, with 2-Coumaroylquinic acid, Myricetin, Rosmarinic Acid, and Quercetin standing out as promising and environmentally friendly alternatives to conventional fungicides. These findings have significant implications for developing crop protection strategies and enhancing global food security, particularly in rice-dependent regions.

## 1. Introduction

Rice is a crucial global staple, especially in Asia. China is the largest producer, responsible for over 90% of the world’s rice and about 30% of global output. India follows closely, producing around 20% of global rice. For impoverished Asians, rice can make up to half of their daily calorie intake, ensuring food security. Rice farming not only supports food security but also plays a vital role in reducing poverty and promoting rural development. Additionally, rice is a significant contributor to Bangladesh’s economy, accounting for almost 20% of GDP and one-sixth of national income.

However, over 70 different diseases affecting rice have been identified, caused by fungi, bacteria, viruses, or nematodes [1]. Bangladesh alone faces 32 specific rice diseases [2]. The devastating rice blast disease, caused by the fungus *Magnaporthe oryzae*, results in significant annual rice losses. In eastern India, approximately 564,000 tons of rice are lost to blast disease each year, with almost half of the losses occurring in upland ecosystems [3]. Although not new to Bangladesh, a severe outbreak of rice blast occurred during the Boro season in 2017 and 2018, affecting several districts [4].

*M*. *oryzae*, belonging to the Magnaporthaceae family and Sordariomycetes class, attacks various parts of the rice plant, including leaves, panicles, stems, and grains [5]. This fungus follows a complex life cycle and exhibits genetic variation that enables it to overcome host defenses [6, 7]. Specific proteins produced by *M*. *oryzae*, known as effectors, play a crucial role in evading the plant’s immune response and establishing successful infections. Key proteins involved in *M*. *oryzae* infection include Catalase-peroxidases 2 (CATALASE PEROXIDASES 2), Hybrid PKS-NRPS synthetase TAS1 (HYBRID PKS-NRPS SYNTHETASE TAS1), Manganese lipoxygenase (MANGANESE LIPOXYGENASE), and Pre-mRNA-splicing factor CEF1 (PRE-MRNA-SPLICING FACTOR CEF1).

HYBRID PKS-NRPS SYNTHETASE TAS1 regulates the production of the toxin tenuazonic acid (TeA), which inhibits protein biosynthesis and photosynthetic processes in rice [8]. Blocking this protein could limit the mechanisms that contribute to rice blast disease [9]. CATALASE PEROXIDASES 2 contributes to the fungal defense against hydrogen peroxide (H2O2) produced by the host during early infection stages [10]. Inhibiting this enzyme can hinder the pathogen’s survival during infection. MANGANESE LIPOXYGENASE complex (Mo-MnLOX) expressed by *M*. *oryzae* plays a vital role in promoting plant necrosis, invasive hyphal development, and infection [11]. Lastly, PRE-MRNA-SPLICING FACTOR CEF1 competes with hnRNP A1, influencing alternative splicing and regulating cell cycle processes [12].

Understanding the functions of these key proteins and their role in *M*. *oryzae*’s infection process is essential for developing strategies to combat rice blast disease. Targeting these proteins could provide promising avenues for intervention and crop protection, reducing the devastating impact of this disease on rice production. Hence, this study focused on evaluating new fungicide candidates derived from natural substances to combat rice blast disease. Natural fungicides can be used effectively by targeting crucial proteins of M. oryzae [13]. Natural fungicides offer advantages such as antifungal activity, aiding plant defense mechanisms, and being environmentally friendly [14]. Various *in silico* analyses, including global molecular docking, high throughput molecular dynamics simulation, and toxicity analysis, were utilized to establish a comprehensive understanding of the interactions between existing fungicides and natural compounds. The primary objective of this study was to identify novel fungicide candidates and examine their detailed interactions with the fungus’s key proteins which can contribute to drug design research and enhance the understanding of molecular interactions to effectively combat rice blast disease on a global scale.

## 2. Methods

**Fig 1:**
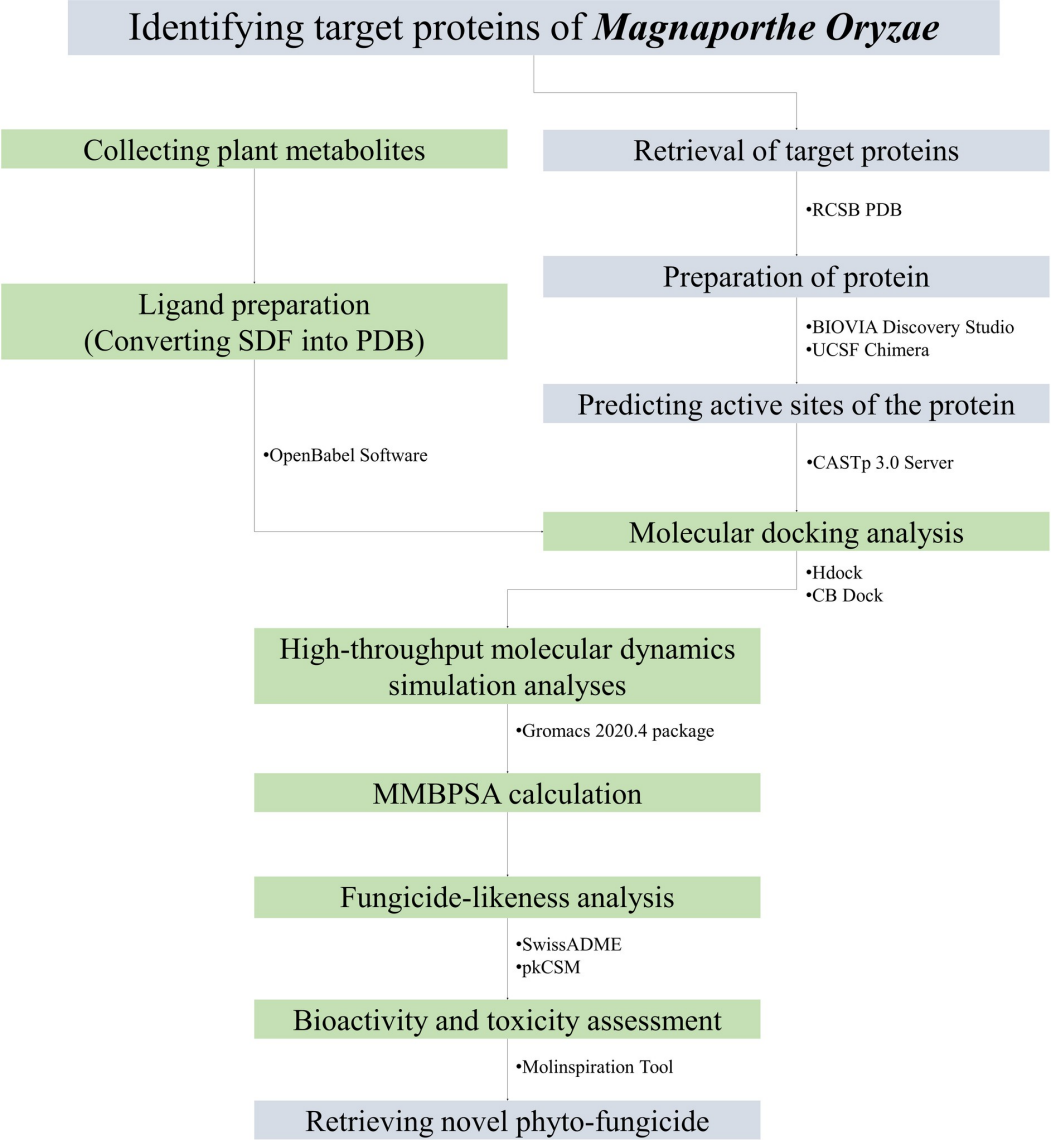
Schematic representation of the methods followed in the whole study.

The methodology for this study involves a well-established protocol for molecular docking and molecular dynamics simulation [13–17]. The stepwise methods used in the study are illustrated in **Fig 1**.

### 2.1. Selection of the target proteins

In this study, four vital proteins essential for the survival of the rice blast fungus M. oryzae were chosen based on a literature review. **S1 Table** provides details about their functions. The RCSB Protein Data Bank (https://www.rcsb.org/) served as a valuable resource for acquiring the structures of these proteins. The Protein Data Bank (PDB) library contains 3D structures of various large biological molecules, including proteins, DNA, and RNA [15, 16]. This comprehensive database plays a crucial role in research and education across multiple fields, including fundamental biology, health, energy, and biotechnology.

### 2.2. Protein preparation

The target protein structure was refined using the BIOVIA Discovery Studio Visualizer program to eliminate undesired ligands, metals, and ions [17]. This software enables visualization, profiling, and analysis of chemical libraries from different sources, assisting in the optimization of compound selection [18]. For protein visualization, UCSF Chimera Software [19] was used.

### 2.3. Ligand preparation

A total of thirty secondary metabolites with established antifungal activity from different plant sources were meticulously chosen through a literature review. These metabolites, substances, and inhibitors show potential as therapeutic candidates against fungi. To evaluate their binding affinities, reference ligands Azoxystrobin, Propiconazole and Tricyclazole were utilized [20–23].

The metabolite structures, available in SDF (3D) format, were obtained from the PubChem database (https://pubchem.ncbi.nlm.nih.gov/) [24]. To prepare them for further analysis, the structures were converted to PDB format using the versatile chemical data tool Open Babel v2.3, which supports over 110 different formats [25].

### 2.4. Active site identification in selected proteins

The CASTp server (http://sts.bioe.uic.edu/castp/) was employed to identify the active sites of the proteins. This server offers a comprehensive map of a protein’s surface topography, precisely identifying and measuring active pockets both on the protein’s surface and within its three-dimensional structure. By utilizing the CASTp server, specific regions and essential residues of the protein that interact with ligands can be predicted with accuracy [26].

### 2.5. Determining metabolite and inhibitor binding affinity

To explore the binding affinity between metabolites and proteins, molecular docking, an essential tool for studying interactions between small ligands and large molecules, was employed. The H-DOCK Server (http://hdock.phys.hust.edu.cn/) was chosen for docking [27], as it provides the necessary tools to discover effective treatments for targeted medicine against deadly pathogens [28]. Furthermore, the CB-Dock server (http://clab.labshare.cn/cb-dock/php/blinddock.php) was used to confirm the binding affinity between metabolites and proteins [29]. CB-Dock utilizes a curvature-based cavity identification approach and Autodock Vina to predict binding sites, calculate their centers and sizes, and evaluate the binding interactions [30].

### 2.6. Examination of the preferred binding location for superior metabolites

For the analysis of binding sites and visualization of results, the PyMOL v2.0 tool was employed. PyMOL is a widely used program for creating high-quality 3D images of biological macromolecules, including proteins and small compounds. It is particularly helpful for visualizing ligand-binding complexes. PyMOL, developed by Warren Lyford DeLano, is a proprietary, open-source molecular visualization tool and one of the primary open-source tools for structural biology visualization. Using PyMOL, polar and non-polar residues were identified, and the binding locations for specific metabolites were examined [31–33].

### 2.7. Molecular dynamics simulation studies

The investigated targets, CATALASE PEROXIDASES 2, HYBRID PKS-NRPS SYNTHETASE TAS1, MANGANESE LIPOXYGENASE, and PRE-MRNA-SPLICING FACTOR CEF1 in complex with the reference standard azoxystobin and the potential target candidates 1-coumarinylqinic acid, rosmarinic acid and myricetin were subjected to 200 ns molecular dynamics (MD) simulation studies to get deeper insights into the possible binding affinities against these targets. The MD simulations were performed using the Gromacs-2020.4 [34, 35] program on the HPC cluster at Bioinformatics Resources and Applications Facility (BRAF), C-DAC, Pune. The requisite topologies of proteins were generated using the CHARMM-36 force field parameters [36, 37]. The complexes were placed in a dodecahedron unit cell and solvated by adding the water using the TIP3P water model [38] keeping the edges of system 1 nm away from the box. The solvated systems were neutralized by adding sodium and chloride counter-ions so as to achieve the molar concentration of 0.15 mole. Further, the systems were energy minimized in order to relieve the steric strains with the steepest descent algorithm until the force-constant reached the threshold of 100 kJ mol^-1^ nm^-1^. Consequently the systems were equilibrated at constant temperature and pressure conditions, NVT and NPT conditions, at 300 K temperature and 1 atm pressure condition using a modified Berendsen thermostat [39] and barostat [40], respectively for 1 ns each. The final 200 ns production phase MD simulation were performed where the temperature conditions of 300 K were achieved with a modified Berendensen thermostat and pressure conditions of 1 atm were achieved with the Parrinello-Rahman barostat [41]. The covalent bonds in the systems were restrained with the LINCS algorithm [42] and the long-range electrostatic energies were computed with Particle Mesh Ewald (PME) method [43] with a cut-off of 1.2 nm. The output of production phase MD simulations was treated for periodic boundary conditions and further analyzed for the root mean square deviations (RMSD) in the backbone atoms of proteins under study, the root mean square fluctuation (RMSF) in the side chain atoms, radius of gyration (Rg), the analysis of solvent accessible solvent area (SASA), and the analysis contact frequency between residues and the ligands. The mean smallest distances between residues pairs, obtained using the gmx mdmat program, were used to construct the contact maps [44]. Further the hydrogen bonds formatted between the binding site residue and ligand were analyzed and the visually inspected in the trajectories at equilibrium state, 50, 100, 150, and 200 ns. The predominant stable conformations existing during MD simulation in each protein were studied through cluster analysis using TTClust program [45]. The major path of motions in each complex was analyzed from principal component analysis (PCA) [46], where the covariance matrix for the C-α atom was diagonalized to obtain the eigenvectors and eigenvalues. The eigenvectors represent the motion path, while eigenvalues represent the mean square fluctuation. The first two eigenvectors, principal components (PC1 and PC2) were further used to obtain the Gibb’s free energy landscape [47]. Molecular mechanics energies combined with Poisson Boltzmann surface area continuum solvation (MM-PBSA) [48, 49] calculations were performed on the trajectories isolated at each 1 ns from 175 to 200 ns.

### 2.8. Analysis of fungicide-like properties

It is important that potential compound to contain biochemical characteristics of good fungicide. That’s why early examination during fungicide discovery can reduce the risk of medication-related failures in later stages. The physicochemical features of the candidate compounds were examined in order to determine their fungicide-like nature. We assessed the natural compounds’ fungicide-likeness using the well-established fundamental rule of drug-likeness (Lipinski’s rule of 5) because there are currently no similar criteria for fungicides [50]. The SwissADME server (http://www.swissadme.ch/) was utilized to assess the fungicide qualities of the top metabolites [51, 52], providing predictions based on the compounds’ characteristics related to Lipinski Rule [53, 54].

### 2.9. Toxicity analysis

The pkCSM web-based server (https://biosig.lab.uq.edu.au/pkcsm/) was used to forecast the general toxicity of the identified compounds [55]. This tool relies on graph-based signatures to analyze molecular distance patterns, making it useful for predicting pharmacokinetic features.

### 2.10. Evaluation of bioavailability and bioactivity score

The canonical SMILES of the ligands obtained from PubChem were subjected to analysis using the Molinspiration online tool (https://www.molinspiration.com/). The properties related to G-protein coupled receptors (GPCR), enzyme inhibitors (EI), kinase inhibitors (KI), nuclear receptor ligands (NRL), and ion channel modulators (ICM) were examined to determine the bioactivity scores [56, 57]. Additionally, the SwissADME tool was employed to quickly establish the bioavailability of the ligands [58].

## 3. Results

### 3.1. Selection and preparation of target proteins

Four crucial proteins that play a vital role in inhibiting M. oryzae, the causative agent of rice blast disease, have been successfully identified through a comprehensive review of the literature. The following selections were retrieved from the Protein Data Bank (PDB) to acquire these significant proteins: Catalase peroxidases 2 (PDB ID 3UT2), Hybrid PKS-NRPS synthetase TAS1 (PDB ID 6KOG), Manganese lipoxygenase (PDB ID 5FNO), and Pre-mRNA-splicing factor CEF1 (PDB ID 6JUI).

To process the obtained proteins, BIOVIA Discovery Studio was utilized, involving the elimination of undesirable macromolecule ligands, water molecules, and heteroatoms from the protein structures. Subsequently, the proteins were subjected to molecular graphics and analyses using UCSF Chimera, as illustrated in **S1A–S1D Fig**.

### 3.2. Prediction of active site of the target proteins

**S1E–S1H Fig** presents the results from the CASTp server, displaying the predicted active binding sites for the selected proteins. The CATALASE PEROXIDASES 2 protein exhibited an Active Site Area (SA) of 979.960 Å^2^ and a Volume (SA) of 922.351 Å^3^. The HYBRID PKS-NRPS SYNTHETASE TAS1 protein displayed an Area (SA) of 2184.725 Å^2^ and a Volume (SA) of 1396.042 Å^3^. For the MANGANESE LIPOXYGENASE protein, the Active Site Area (SA) was found to be 3294.823 Å^2^ with a Volume (SA) of 2471.753 Å^3^, while the PRE-MRNA-SPLICING FACTOR CEF1 protein showed an Area (SA) of 176.379 Å^2^ and a Volume (SA) of 255.012 Å^3^. The predicted active site information for each of the proteins can be found in **S2 Table**.

### 3.3. Ligand preparation

A comprehensive review of existing literature was conducted to gather a list of 30 metabolites (**S3 Table**) derived from various plants that exhibit antifungal properties. These metabolites, along with inhibitors and compounds, hold potential as therapeutic candidates for combating diverse fungal infections. The 3D structures of all 30 metabolites were acquired in SDF format from the PubChem database. Using the Open Babel v2.3 software, these structures were converted into the PDB format. The same procedure was applied to obtain the PDB format for the reference compounds Azoxystrobin and Tricyclazole.

### 3.4. Molecular docking studies

Molecular docking experiments were conducted, with the selected proteins used as receptors and the 30 plant metabolites (**S4 Table**) employed as ligands. Among the ligands tested, namely 2-Coumaroylquinic acid, Myricetin, Rosmarinic Acid, and Quercetin, superior performance was observed in terms of binding to the target proteins. Notably, the target proteins were bound more effectively by these metabolites compared to the reference ligands Azoxystrobin, Propiconazole and Tricyclazole. In the H-Dock docking analysis, the highest binding affinities against the MANGANESE LIPOXYGENASE protein (-211.31) and the HYBRID PKS-NRPS SYNTHETASE TAS1 protein (-198.49) were exhibited by Rosmarinic acid. For the PRE-MRNA-SPLICING FACTOR CEF1 protein, Myricetin displayed the highest binding affinity (-143.36), while 2-Coumaroylquinic acid showed the highest binding affinity against the CATALASE PEROXIDASES 2 protein (-206.22). All the reference drug Azoxystrobin, Propiconazole and Tricyclazole showed almost similar type of docking score, among them Azoxystrobin showed a higher affinity.

**Fig 2:**
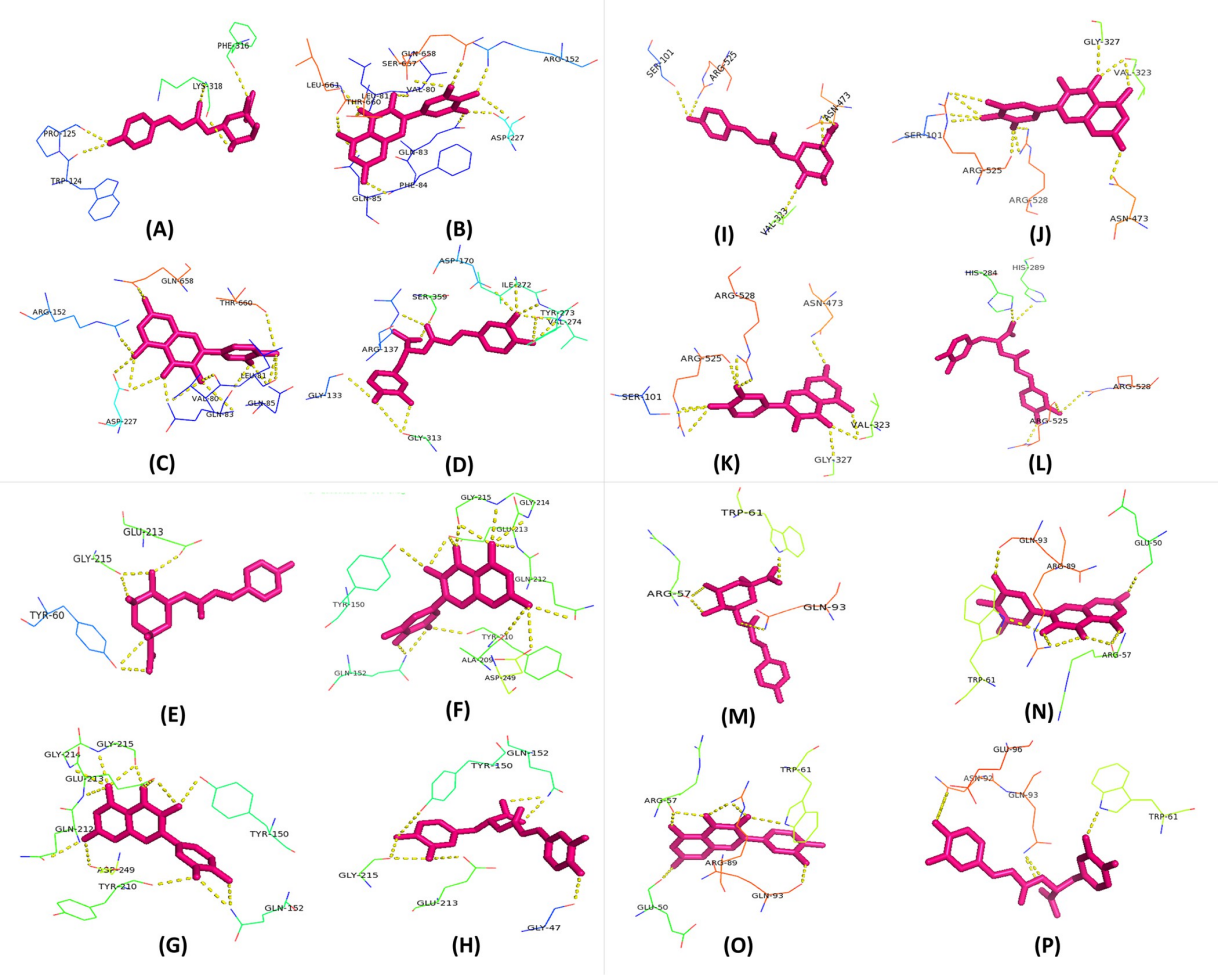
Interaction of polar binding site residues in CATALASE PEROXIDASES 2, HYBRID PKS-NRPS SYNTHETASE TAS1, MANGANESE LIPOXYGENASE, and PMSF proteins with various compounds: A) 2-Coumaroylquinic acid, B) Myricetin, C) Quercetin, and D) Rosmarinic Acid for CATALASE PEROXIDASES 2; E) 2-Coumaroylquinic acid, F) Myricetin, G) Quercetin, and H) Rosmarinic Acid for HYBRID PKS-NRPS SYNTHETASE TAS1; I) 2-Coumaroylquinic acid, J) Myricetin, K) Quercetin, and L) Rosmarinic Acid for MANGANESE LIPOXYGENASE; and M) 2-Coumaroylquinic acid, N) Myricetin, O) Quercetin, and P) Rosmarinic Acid for PMSF.

Overall, greater affinity in the H-Dock analysis was demonstrated by Rosmarinic acid and Myricetin, and all four of these top metabolites displayed superior binding affinity compared to the reference ligands. The predictions were further supported by the CB Dock analysis. Notably, the highest polar binding site with the CATALASE PEROXIDASES 2 protein was shown by Myricetin in the evaluation using PyMol software. The binding sites, as mentioned in **Table 1** and **Fig 2**, included LEU-661, THR-660, LEU-81, VAL-80, SER-657, GLN-658, ARG-152, ASP-227, GLN-83, PHE-84, and GLN-85. The best metabolites 1-coumarinylqinic acid, rosmarinic acid, and myricetin based on binding affinities were evaluated in further molecular dynamic simulation studies.

**Table 1:** Binding sites of best metabolites with target protein.

Protein Name	Ligand	HDock		CB Dock	Polar Binding Site
		Docking Score	Confidence score	Vina Score	
MANGANESE LIPOXYGENASE	Rosmarinic acid	-211.31	0.7732	-9.6	HIS-284, HIS-289, ARG-525, ARG-528
	Myricetin	-204.42	0.7481	-9.6	SER-101, ARG-525, ARG-528, ASN-473, VAL-323, GLY-327
	2-Coumaroylquinic acid	-203.47	0.7445	-8.6	SER-101, ARG-525, ASN-473, VAL-323
	Quercetin	-196.15	0.7157	-9.6	SER-101, ARG-525, ARG-528, ASN-473, VAL-323, GLY-327
	**Azoxystrobin (Ref Drug)**	**-176.87**	**0.6312**	**-8.4**	**ARG-528, ARG-525**
	**Tricyclazole (ref Drug)**	**-96.46**	**0.2553**	**-7.4**	**PHE-240**
	**Propiconazole (Ref Drug)**	**-152.77**	**0.5138**	**-8.1**	**ASN-473**
PRE-MRNA-SPLICING FACTOR CEF1	Myricetin	-143.36	0.4668	-6.7	GLN-93, ARG-89, GLU-50, ARG-57, TRP-61
	Rosmarinic acid	-142.90	0.4646	-7	ASN-92, GLU-96, GLN-93, TRP-61
	Quercetin	-141.30	0.4566	-6.6	ARG-57, ARG-89, GLN-93, GLU-50, TRP-61
	2-Coumaroylquinic acid	-140.56	0.4529	-6.3	ARG-57, TRP-61, GLN-93
	**Azoxystrobin (Ref Drug)**	**-137.86**	**0.4396**	**-6.8**	**ARG-89, ARG-47**
	**Tricyclazole (Ref Drug)**	**-73.55**	**0.1781**	**-5.3**	**ARG-89**
	**Propiconazole (Ref Drug)**	**-108.35**	**0.3030**	**-5.6**	**GLN-93**
CATALASE PEROXIDASES 2	2-Coumaroylquinic acid	-206.22	0.7548	-9	PRO-125, TRP-124, LYS-318, PHE-316
	Rosmarinic acid	-196.92	0.7188	-9.3	GLY-133, ARG-137, SER-359, ASP-170, ILE-272, TYR-273, VAL-274
	Myricetin	-190.29	0.6912	-10.1	LEU-661, THR-660, LEU81, VAL-80, SER-657, GLN-658, ARG-152, ASP-227, GLN-83, PHE-84, GLN-85
	Quercetin	-181.77	0.6537	-10.6	ARG-152, ASP-227, GLN-83, VAL-80, GLN-85, LEU-81, THR-660, GLN-658
	**Azoxystrobin (Ref Drug)**	**-194.55**	**0.7091**	**-8.9**	**HIS-141, SER-359**
	**Tricyclazole (Ref drug)**	**-101.28**	**0.2740**	**-7.1**	**ARG-179**
	**Propiconazole (Ref Drug)**	**-146.56**	**0.4828**	**-8.1**	**THR-273**
HYBRID PKS-NRPS SYNTHETASE TAS1	Rosmarinic acid	-198.49	0.7251	-9.7	GLY-215, GLU-213, GLY-47, TYR-150, GLN-152
	Myricetin	-195.25	0.7120	-9.3	TYR-150, GLN-152, ASN-209, ASP-249, ALA-209, TYR-210, GLN-212, GLU-213, GLY-214, GLY-215
	Quercetin	-186.41	0.6744	-9.4	GLY-214, GLU-213, GLN-212, ASP-249, TYR-210, GLN-152, TYR-150, GLY-215
	2-Coumaroylquinic acid	-174.15	0.6185	-9	TYR-60, GLY-215, GLU-213
	**Azoxystrobin (Ref Drug)**	**-176.47**	**0.6293**	**-7.9**	**SER-324, GLY-290**
	**Tricyclazole (Ref Drug)**	**-95.79**	**0.2527**	**-6.7**	**THR-326**
	**Propiconazole (Ref drug)**	**-141.81**	**0.4591**	**-6.9**	**SER-324**

### 3.5. Molecular dynamics simulation studies

#### 3.5.1. Root mean square deviation

**Fig 3:**
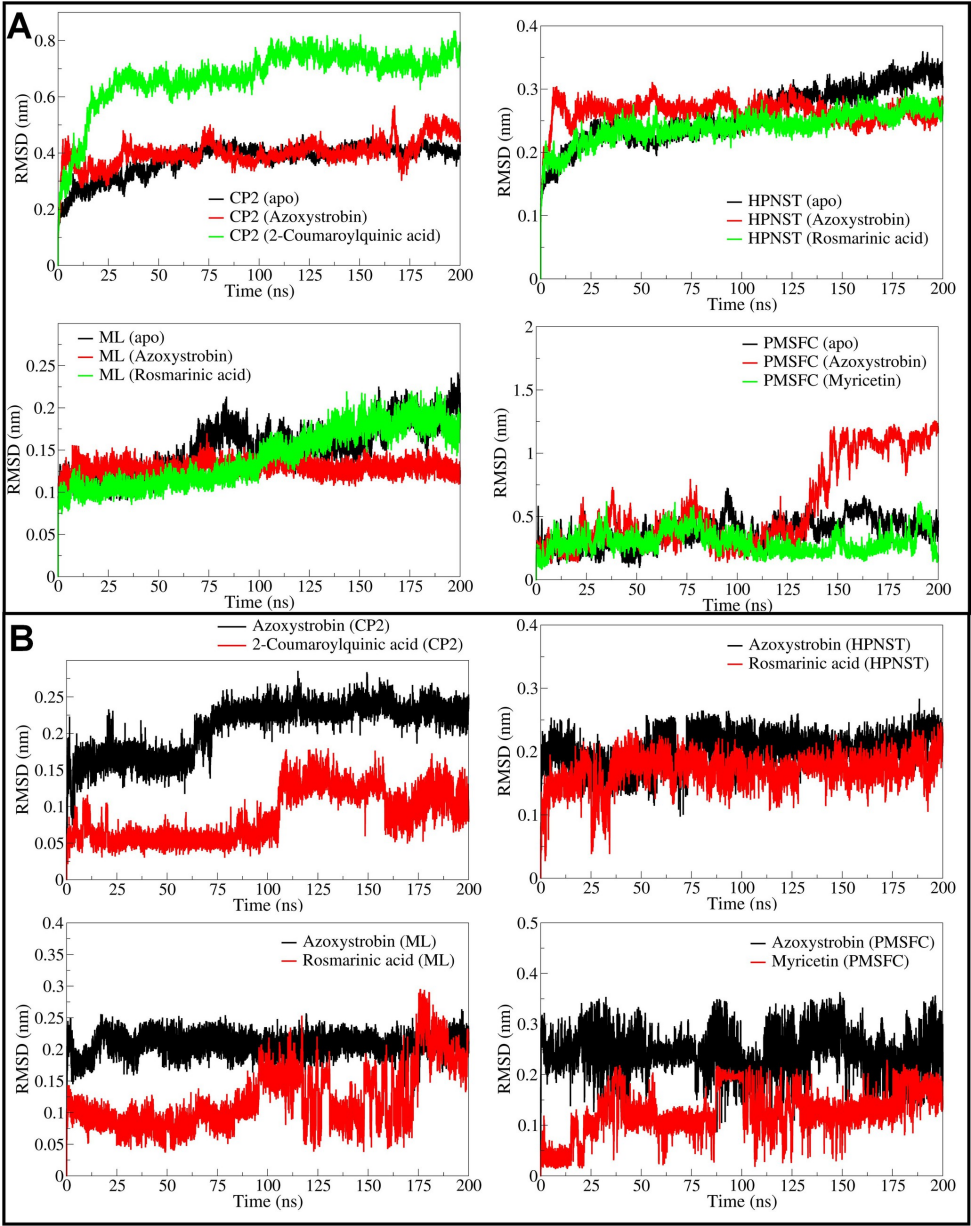
RMSD analysis of backbone atoms in CATALASE PEROXIDASES 2, HYBRID PKS-NRPS SYNTHETASE TAS1, MANGANESE LIPOXYGENASE, and PMSF proteins (A), and RMSD analysis of azoxystrobin, 2-coumaroylquinic acid, and rosmarinic acid in respective complexes (B).

The RMSD in the backbone atoms of apo CATALASE PEROXIDASES 2 was found reasonably stable with an average of 0.3458 nm (**Fig 3A** and **Table 2**). The RMSD in CATALASE PEROXIDASES 2 backbone atoms in 2-coumaroylquinic acid bound complex was found to be larger compared to the azoxystrobin bound form with an average of 0.6065 and 0.3796 nm respectively in these cases. However, the RMSD in atoms of ligands bound to CATALASE PEROXIDASES 2, for instance the 2-coumaroylquinic acid is lower than the azoxystrobin with an average of 0.05755 and 0.1831 nm, respectively (**Fig 3B**). In the case of HYBRID PKS-NRPS SYNTHETASE TAS1, the RMSD in backbone atoms of apo form and rosmarinic acid bound form is almost similar until around 100 ns simulation period. However, the RMSD in backbone atoms of HYBRID PKS-NRPS SYNTHETASE TAS1 bound with rosmarinic acid remained stable thereafter with an average of 0.2253 nm. The RMSD in backbone atoms of HYBRID PKS-NRPS SYNTHETASE TAS1 bound with azoxystrobin was found slightly higher with an average of 0.2659 nm. Compared to azoxystrobin bound form and apo form, the RMSD in backbone atoms of HYBRID PKS-NRPS SYNTHETASE TAS1 bound to rosmarinic acid is lower and reasonably stable with an average of 0.2253 nm. Further, the RMSD in rosmarinic acid atoms is also lower compared to azoxystrobin with an average of 0.1648 nm. The RMSD in backbone atoms of MANGANESE LIPOXYGENASE bound to rosmarinic acid is lowest until 100 ns simulation period and thereafter rises to equaling the RMSD in backbone atoms of apo form. The overall average RMSD for backbone atoms of rosmarinic acid bound form of MANGANESE LIPOXYGENASE is lowest with an average of 0.1111 nm compared to azoxystrobin bound form with an average of 0.1277 nm and apo form with an average of 0.1322 nm. The RMSD in rosmarinic acid atoms is significantly lower compared to atoms of azoxystrobin with an average of 0.0930 nm. In the case of PRE-MRNA-SPLICING FACTOR CEF1 the RMSD in backbone atoms was lowest with myricetin bound form with an average of 0.322 nm, compared to apo form for which the average is 0.3250 nm. The azoxystrobin bound form has slightly higher RMSD in backbone atoms with an average of 0.3516 nm. The RMSD in atoms of azoxystrobin is higher with an average of 0.2472 nm compared to myricetin which has an average RMSD of 0.1117 nm.

**Table 2:** Estimates of averages for different MDS analysis parameters.

Details of the complexes	Average (nm)				
	RMSD in backbone atoms	RMSD in UNK atoms	RMSF	Gyrate	SASA
CATALASE PEROXIDASES 2 (apo)	0.3458 (0.0656)	-	0.1670 (0.1416)	2.8406 (0.0107)	322.9365 (4.7790)
CATALASE PEROXIDASES 2 (Azoxystrobin)	0.3796 (0.0419)	0.1831 (0.0333)	0.1854 (0.1626)	2.8545 (0.0138)	321.6773 (4.6042)
CATALASE PEROXIDASES 2 (1-Coumaroylquinic acid)	0.6065 (0.1155)	0.05755 (0.0113)	0.1859 (0.1519)	2.9124 (0.0236)	329.2893 (5.5271)
HYBRID PKS-NRPS SYNTHETASE TAS1 (apo)	02215 (0.0250)	-	0.1140 (0.0825)	2.1800 (0.0072)	322.6027 (4.8412)
HYBRID PKS-NRPS SYNTHETASE TAS1 (Azoxystrobin)	0.2659 (0.02217)	0.1944 (0.0296)	0.1151 (0.0851)	2.1714 (0.0067)	325.6557 (5.3237)
HYBRID PKS-NRPS SYNTHETASE TAS1 (Rosmarinic acid)	0.2253 (0.0220)	0.1648 (0.0292)	0.1111 (0.0787)	2.1351 (0.0077)	319.1804 (5.3598)
MANGANESE LIPOXYGENASE (apo)	0.1322 (0.0254)	-	0.1112 (0.0608)	2.4601 (0.0080)	228.1651 (4.1517)
MANGANESE LIPOXYGENASE (Azoxystrobin)	0.1277 (0.0088)	0.2074 (0.0175)	0.1031 (0.0518)	2.4678 (0.0061)	231.9011 (2.9584)
MANGANESE LIPOXYGENASE (Rosmarinic acid)	0.1111 (0.0113)	0.0930 (0.0235)	0.1124 (0.0578)	2.4578 (0.0071)	229.2028 (3.5457)
PRE-MRNA-SPLICING FACTOR CEF1 (apo)	0.3250 (0.1023)	-	0.2948 (0.1207)	1.6117 (0.0626)	75.2769 (1.6012)
PRE-MRNA-SPLICING FACTOR CEF1 (Azoxystrobin)	0.3516 (0.1172)	0.2472 (0.0319)	0.5843 (0.1857)	1.6853 (0.0511)	76.8776 (2.0944)
PRE-MRNA-SPLICING FACTOR CEF1 (Myricetin)	0.322 (0.0822)	0.1117 (0.04987)	0.2571 (0.0992)	1.6667 (0.0360)	76.9609 (2.0344)

Standard deviations in average values are given in parentheses

#### 3.5.2. Root mean square fluctuation

**Fig 4:**
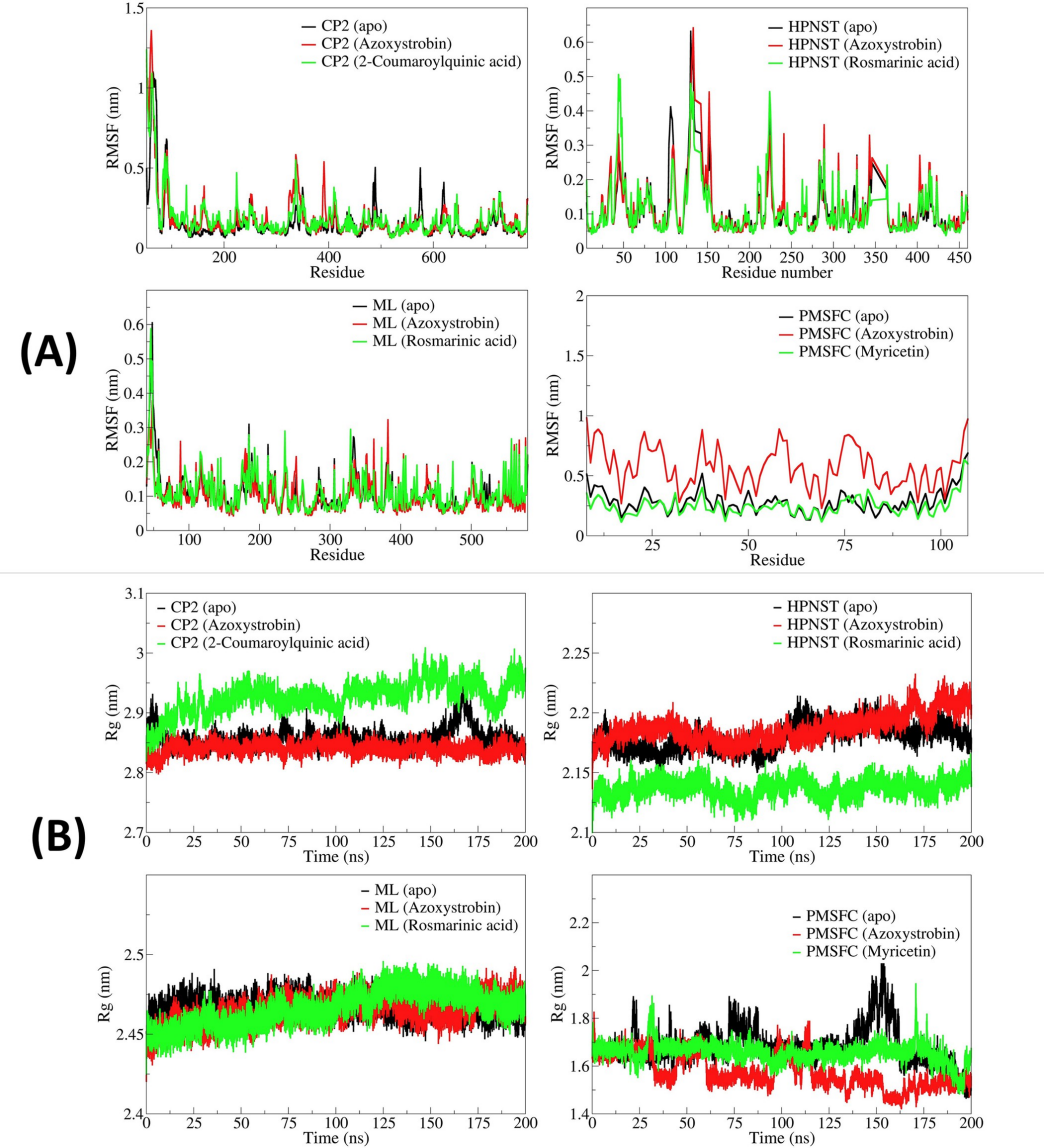
(A) RMSF in side chain atoms of residues in CATALASE PEROXIDASES 2, HYBRID PKS-NRPS SYNTHETASE TAS1, MANGANESE LIPOXYGENASE, and PMSF proteins, and (B) Radius of gyration for corresponding systems of CATALASE PEROXIDASES 2, HYBRID PKS-NRPS SYNTHETASE TAS1, MANGANESE LIPOXYGENASE, and PMSF.

In the case of CATALASE PEROXIDASES 2-complexes, the apo CATALASE PEROXIDASES 2 showed comparably lower RMSF in the side chain atoms of the residues with an average RMSF of 0.1670 nm (**Fig 4A**). The RMSF remained almost similar in the case of azoxystrobin and 2-coumaroylquinic acid bound CATALASE PEROXIDASES 2 with an average of 0.1854 and 0.1859 nm, respectively. In the case of HYBRID PKS-NRPS SYNTHETASE TAS1, the rosmarinic acid bound form showed lower RMSF (average 0.1111 nm) compared to apo HYBRID PKS-NRPS SYNTHETASE TAS1 (average 0.1140 nm) and azoxystrobin bound HYBRID PKS-NRPS SYNTHETASE TAS1 (average 0.1151). The RMSF in azoxystrobin bound MANGANESE LIPOXYGENASE was found lowest with an average of 0.1031 nm compared to the apo MANGANESE LIPOXYGENASE having an average RMSF of 0.1112 nm and rosmarinic acid bound MANGANESE LIPOXYGENASE which has an average RMSF of 0.1124 nm. In the case of PRE-MRNA-SPLICING FACTOR CEF1, myricetin bound form of PRE-MRNA-SPLICING FACTOR CEF1 has the lowest RMSF with an average of 0.2571 nm. The apo PRE-MRNA-SPLICING FACTOR CEF1 and azoxystrobin bound PRE-MRNA-SPLICING FACTOR CEF1 has shightly larger magnitude of RMSF with averages of 0.2948 and 0.5843 nm respectively.

#### 3.5.3. Radius of gyration

The apo CATALASE PEROXIDASES 2 and azoxystrobin bound CATALASE PEROXIDASES 2 showed the lower total Rg with averages of 2.8406 and 2.8545 nm respectively (**Fig 4B**). While the 1-coumaroylquinic acid bound CATALASE PEROXIDASES 2 showed slightly higher magnitude of Rg with an average of 2.9124 nm. In the case of HYBRID PKS-NRPS SYNTHETASE TAS1 rosmarinic acid bound form showed the lowest Rg with an average of 2.1351 nm. The apo HYBRID PKS-NRPS SYNTHETASE TAS1 and azoxystrobin bound HYBRID PKS-NRPS SYNTHETASE TAS1 showed higher Rg with averages of 2.1800 and 2.1714 nm respectively. Similarly, the rosmarinic acid bound MANGANESE LIPOXYGENASE also showed lower Rg with an average of 2.4578 nm, compared to apo MANGANESE LIPOXYGENASE with an average of 2.4601 nm and azoxystrobin bound MANGANESE LIPOXYGENASE with an average of 2.4678 nm. The apo PRE-MRNA-SPLICING FACTOR CEF1 showed comparably lower Rg with an average of 1.6117 nm than the myricetin bound PRE-MRNA-SPLICING FACTOR CEF1 with an average of 1.6667 nm and azoxystrobin bound PRE-MRNA-SPLICING FACTOR CEF1 with an average of 1.6853 nm.

#### 3.5.4. Solvent accessible surface area analysis

**Fig 5:**
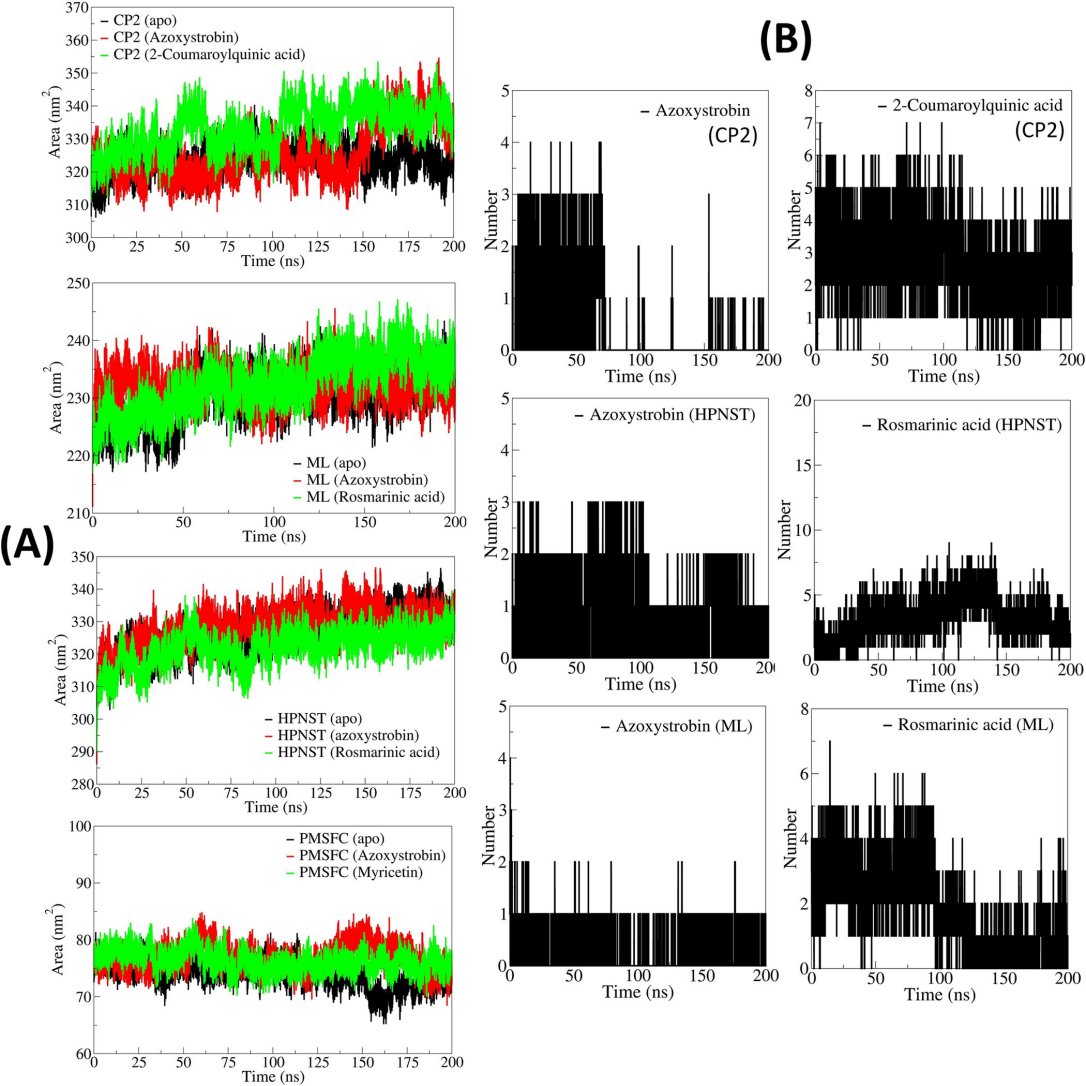
(A) Solvent accessible surface area analysis, and (B) Hydrogen bond analysis.

The solvent accessible surface area (SASA) for azoxystrobin bound CATALASE PEROXIDASES 2 was found lowest with an average of 321.6773 nm^3^ (**Fig 5A**). The apo CATALASE PEROXIDASES 2 and 2-coumaroylquinic acid bound CATALASE PEROXIDASES 2 showed slightly larger SASA with averages of 322.9365 and 329.2893 nm^3^. The SASA for rosmarinic acid bound HYBRID PKS-NRPS SYNTHETASE TAS1 was found lowest with an average of 319.1804 nm^3^. While the SASA for apo HYBRID PKS-NRPS SYNTHETASE TAS1 and azoxystrobin bound HYBRID PKS-NRPS SYNTHETASE TAS1 was slightly higher with averages of 322.6027 and 325.6557 nm^3^ respectively. In the case of MANGANESE LIPOXYGENASE, the apo MANGANESE LIPOXYGENASE showed lowest SASA with an average of 231.9011 nm^3^ compared to the rosmarinic acid bound MANGANESE LIPOXYGENASE with an average of 229.2028 nm^3^ and azoxystrobin bound MANGANESE LIPOXYGENASE with an average of 231.9011 nm^3^. The SASA for apo PRE-MRNA-SPLICING FACTOR CEF1 was lowest with an average of 75.2769 nm^3^, whereas the SASA for azoxystrobin and myricetin bound PRE-MRNA-SPLICING FACTOR CEF1 was almost similar with averages of 76.8776 and 76.9609 nm^3^ respectively.

#### 3.5.5. Hydrogen bond and contact frequency analysis

In the case of CATALASE PEROXIDASES 2 azoxystrobin formed 3 hydrogen bonds until around 60 ns simulation period and thereafter only one hydrogen bond was seen formed occasionally until the end of simulation (**Fig 5B**). While in the case of 2-coumaroylquinic acid 5 hydrogen bonds were seen formed consistently until around 100 ns and thereafter around 4 hydrogen bonds were seen consistently formed until the end of simulation period. However, azoxystrobin, in the case of HYBRID PKS-NRPS SYNTHETASE TAS1 formed 2 consistent hydrogen bonds throughout the simulation period. Rosmarinic acid formed around 5 consistent hydrogen bonds until first 100 ns simulation period and thereafter for a brief period of 50 ns the number of hydrogen bonds rose to around 7. The last 50 ns simulation period showed around 5 hydrogen bonds in this complex. In the case of complexes of MANGANESE LIPOXYGENASE, azoxystrobin showed one hydrogen bond consistently formed throughout the simulation period whereas the rosmarinic acid formed around 4 hydrogen bonds until the first 100 ns simulation period which is lowered to 2 hydrogen bonds thereafter. In the case of PRE-MRNA-SPLICING FACTOR CEF1 neither azoxystrobin nor myricetin could form consistent hydrogen bonds.

**Fig 6:**
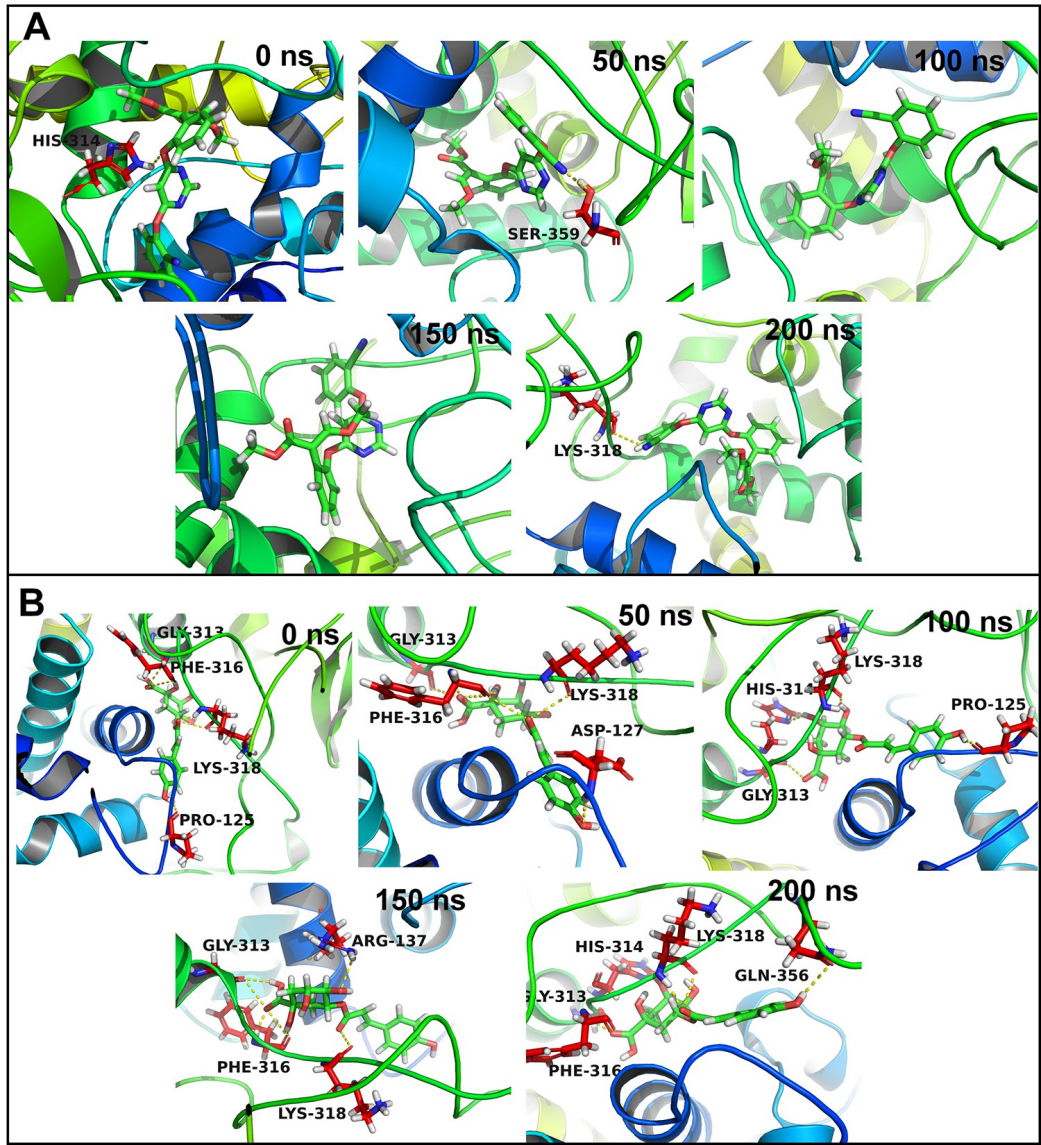
Hydrogen bonds between CATALASE PEROXIDASES 2 and (A) Azoxystrobin and (B) 2-Coumaroylquinic acid.

The residues which can make frequent contacts with the ligands under study were analyzed. In the case of CATALASE PEROXIDASES 2, 24 residues were with 10 Å distance from the azoxystrobin (**S2 Fig**). Out of these residues His141 has the highest contact frequency of 75%. The contact frequency for residues His314, Lys318, Arg137, and Trp365 was more than 50%. In the case of complex of CATALASE PEROXIDASES 2 with 2-coumaroylquinic acid, 23 residues were within 10 Å distance from 2-coumaroylquinic acid. The residues Phe316, Lys318, Gly313, His314, and Gly317 showed contact frequency of over 75%. The trajectories isolated at equilibrated state, and at 50, 100, 150 and 200 ns were analyzed in each complex. The complex of CATALASE PEROXIDASES 2 with azoxystrobin at equilibrated state showed the hydrogen bond with His314 (**Fig 6**). The trajectory isolated at 50 ns state showed a hydrogen bond with Ser359. No hydrogen bonds were seen in the trajectories isolated at 100 and 150 ns. The trajectory at 200 ns showed a hydrogen bond with Lys318. In the case of complex of CATALASE PEROXIDASES 2 with 2-coumaroylquinic acid the trajectory at equilibrated state showed hydrogen bonds with residues Gly313, Phe316, Lys318, and Pro125. Out of these hydrogen bonds the bonds with Gly313, Phe316, and Lys318 almost remained intact as seen in the trajectories at 50, 100, 150 and 200 ns. Apart from these hydrogen bonds the trajectory at 50 ns showed a hydrogen bond with Asp127, the 100 ns trajectory showed with His314, and Pro125, the 150 ns trajectory showed with Arg137, and 200 ns trajectory showed with His314, and Gln356.

**Fig 7:**
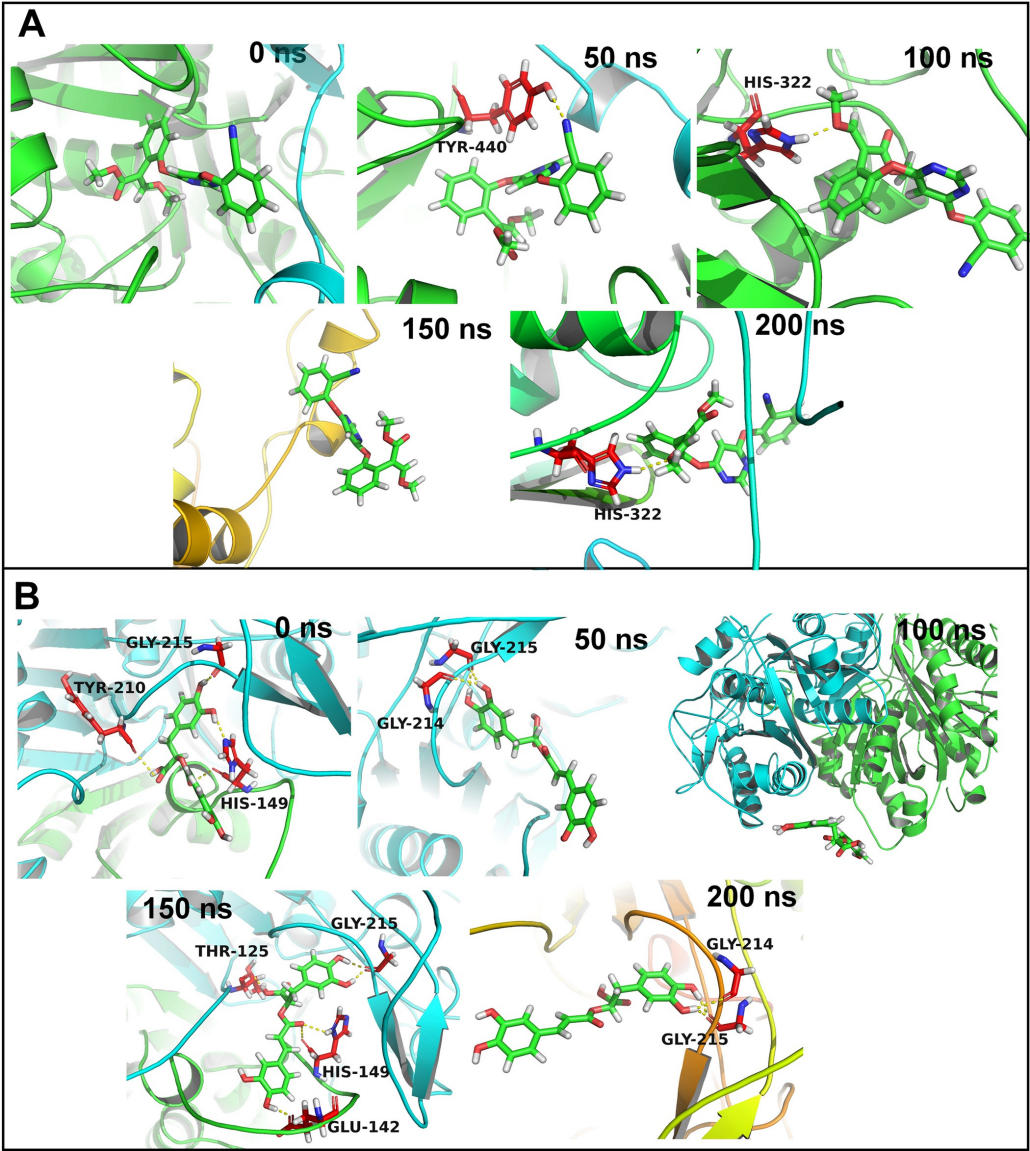
Hydrogen bonds between HYBRID PKS-NRPS SYNTHETASE TAS1 and (A) Azoxystrobin and (B) Rosmarinic acid.

In the case of HYBRID PKS-NRPS SYNTHETASE TAS1 28 residues were within 10 Å distance from the azoxystrobin. None of the residue could form more than 75% contacts with azoxystrobin (**S3 Fig**). The contact frequency for the residues His322, Ser324, Val228, Leu289, and Gly290 was found to be more than 50%. On the other hand, rosmarinic acid has 25 residues within 10 Å distance. Out of these His149, Gly215, and Gly214 showed contact frequency of over 75%. The residues Gln151 and Pro211 showed contact frequency of over 50%. No hydrogen bonds were seen in the equilibrated trajectory and trajectory at 150 ns for HYBRID PKS-NRPS SYNTHETASE TAS1 azoxystrobin complex (**Fig 7**). The trajectory at 50 ns showed a hydrogen bond with Tyr440 and the trajectories at 100, and 200 ns showed a hydrogen bond with His322. The equilibrated trajectory of HYBRID PKS-NRPS SYNTHETASE TAS1 with bound rosmarinic acid showed hydrogen bonds with Gly215, Tyr210, and His149. The hydrogen bond with Gly215 was seen intact in trajectories of 50, 100, 150, and 200 ns. Further, the 50 ns and 200 ns trajectories showed a hydrogen bond with Gly214 and trajectory at 150 ns showed a hydrogen bond with His149 and Glu142.

**Fig 8:**
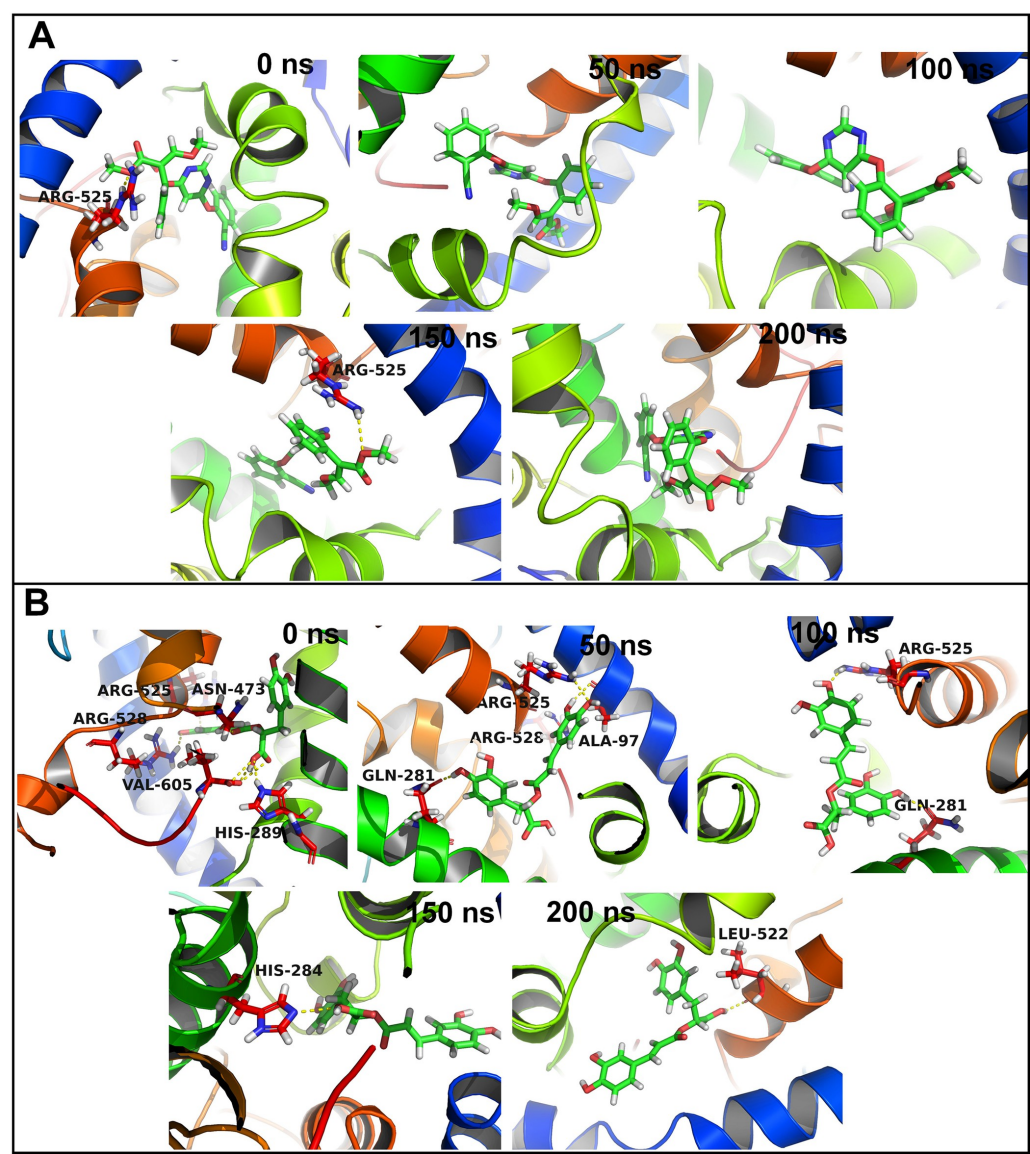
Hydrogen bonds between MANGANESE LIPOXYGENASE and (A) Azoxystrobin and (B) Rosmarinic acid.

In the case of MANGANESE LIPOXYGENASE 18 residues were within 10 Å distance from the azoxystrobin. The residue Arg525 formed more than 75% contacts and residue Val605 formed more than 50% contacts with azoxystrobin (**S4 Fig**). The residues Phe526, Leu331, and Trp93 showed slightly lower contact frequency of 25 to 50%. The equilibrated and trajectory at 150 ns showed a hydrogen bond with Arg525 (**Fig 8**). No hydrogen bonds were found in 50, 100, and 200 ns trajectories. In the case of complex of MANGANESE LIPOXYGENASE with rosmarinic acid the residue Arg525 showed more than 75% contact frequency. The residues Gln281, Ser101, His284, and Phe526 showed just over 50% contact frequency. The equilibrated trajectory showed hydrogen bonds with Arg525, Arg528, Val605, His289, and Asn473. The hydrogen bonds with Arg525 and Arg526 remained intact as seen in 50 ns trajectory along with new hydrogen bonds with Gln281 and Ala97. The 100 ns trajectory showed hydrogen bonds with Arg525 and Gln281. The 150 and 200 ns trajectories showed a hydrogen bond with His284 and Leu522 respectively.

**Fig 9:**
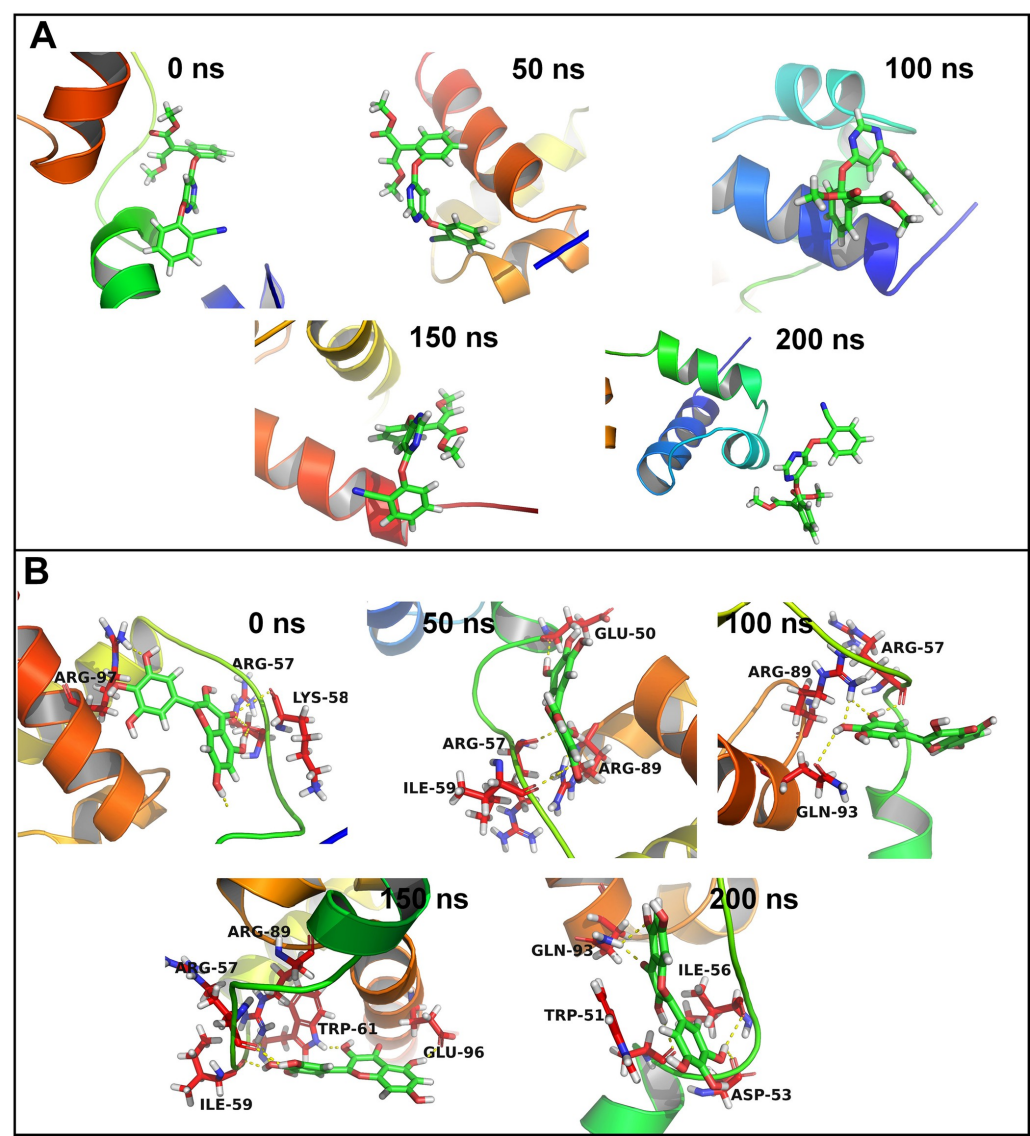
Hydrogen bonds between PMSF and (A) Azoxystrobin and (B) Rosmarinic acid.

In the case of PRE-MRNA-SPLICING FACTOR CEF1 no residues were within 10 Å distance from azoxystrobin and no residues showed any significant contacts with azoxystrobin (**S5 Fig**). However, few residues such as Trp80, Gln99, Tyr98, Lys58, and Leu102 showed around 10% contact frequency. Similarly, the trajectories isolated at equilibrium state, 50, 100, 150 and 200 ns showed no hydrogen bond interactions (**Fig 9**). Myricetin on the other hand has 17 residues within 10 Å distance. The residue Lys58 and Trp61 formed more than 75% contacts, while Ile59, Arg89, and Glu60 formed more than 50% contacts. The equilibrated trajectory of complex of PRE-MRNA-SPLICING FACTOR CEF1 with myricetin showed hydrogen bonds with Arg97, Arg57, and Lys58. The 50 ns trajectory showed hydrogen bonds with Arg57, Ile59, Arg89, and Glu50. The hydrogen bonds with Arg57 and Arg89 remained intact in 100 ns trajectory and a new hydrogen bond with Gln93 formed. The 150 ns trajectory showed hydrogen bonds with Trp61, Arg57, Ile59, Arg89, and Glu96. The 200 ns trajectory showed hydrogen bonds with Gln93, Trp51, Ile56, and Asp53.

#### 3.5.6. Gibb’s free energy analysis

**Fig 10:**
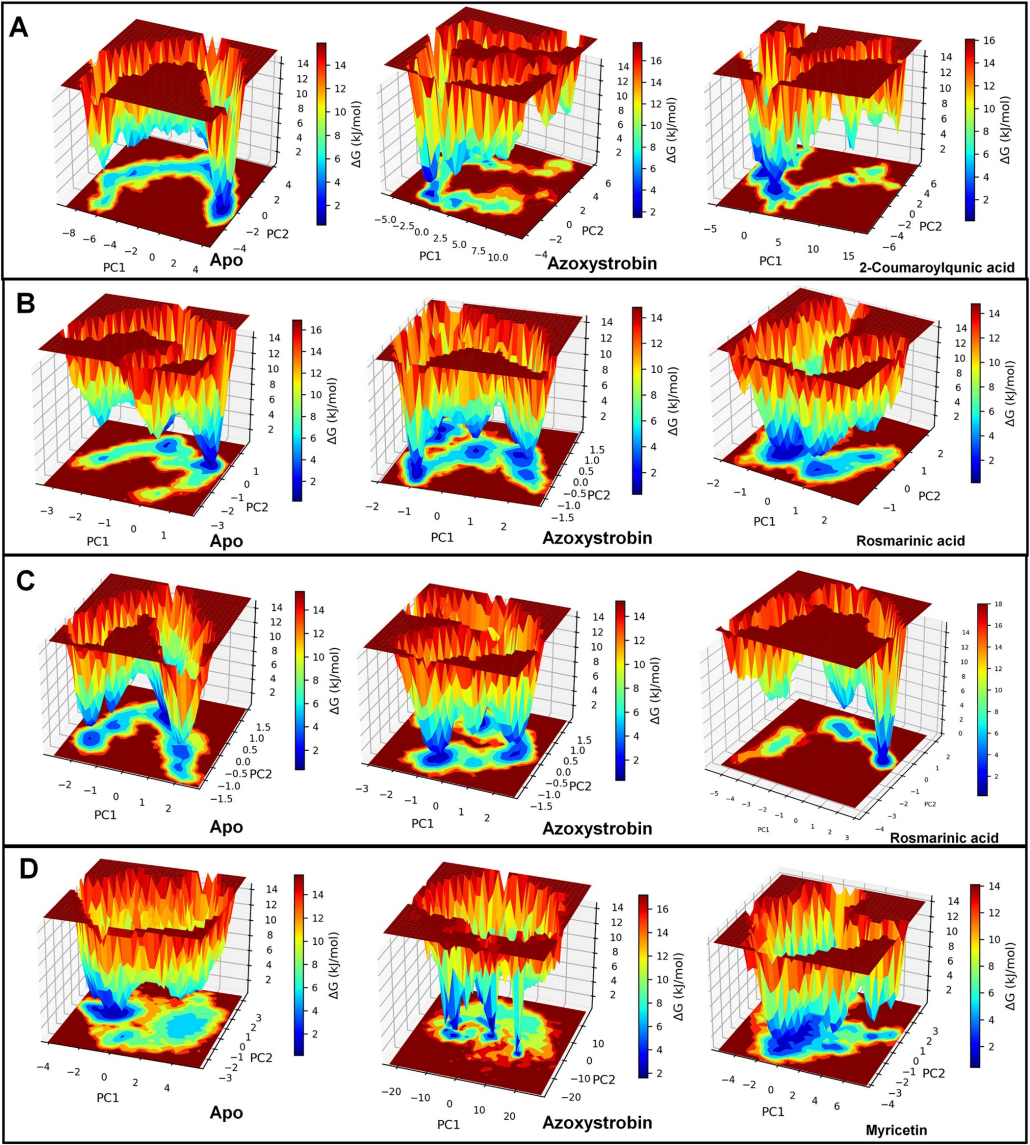
Gibbs’ free energy landscape: (A) Apo CATALASE PEROXIDASES 2 and respective CATALASE PEROXIDASES 2 complexes, (B) Apo HYBRID PKS-NRPS SYNTHETASE TAS1 and respective HYBRID PKS-NRPS SYNTHETASE TAS1 complexes, (C) Apo MANGANESE LIPOXYGENASE and respective MANGANESE LIPOXYGENASE complexes, and (D) Apo PMSF and respective PMSF complexes.

The Gibb’s free energy analysis for apo CATALASE PEROXIDASES 2 showed the lowest energy conformations between 2 to 4 on PC1 and between 0 to -2 on PC2 **(Fig 10A**). In the case of CATALASE PEROXIDASES 2 azoxystrobin complex two small energy basins were found one occupying -2 to -3 on PC1 and 2 to 3 on PC2 and second occupying -2 to -3 on PC1 and -2 to -3 on PC2. Compared to apo CATALASE PEROXIDASES 2 and azoxystrobin bound CATALASE PEROXIDASES 2, the 2-coumaroylquinic acid CATALASE PEROXIDASES 2 complex showed two big energy basins one occupying between 1 to -1 on PC1 and -2 to -4 on PC2 and other occupying -1 to -4 on PC1 and 2 to -1 on PC2.

In the case of apo HYBRID PKS-NRPS SYNTHETASE TAS1 the low energy conformations were found in a small energy basin occupying 0.7 to 1.4 on PC1 and 0.5 to -0.5 on PC2 (**Fig 10B**). Four small energy basins were found for HYBRID PKS-NRPS SYNTHETASE TAS1 azoxystrobin complex. Out of these the non-correlating low energy conformations were found in the energy basin occupying around 0 on PC1 and PC2, while the positively correlating low energy conformations were found in the energy basin occupying 1 to 2 on PC1 and 0 to 0.5 on PC2. Anti-correlating low energy conformations were found in the energy basins one occupying -1 to -1.2 on PC1 and 0.5 to 1 on PC2 and other occupying -1 to -1.5 on PC1 and -0.5 to -1.5 on PC2. The HYBRID PKS-NRPS SYNTHETASE TAS1 rosmarinic acid complex showed low energy conformations in a large energy basin occupying 0 to -1 on PC1 and 0 to -1 on PC2.

In the case of apo MANGANESE LIPOXYGENASE the low energy conformations were found in a energy basin occupying 0 to 1 on PC1 and 0.5 to 1 on PC2 (**Fig 10C**). Two energy basins were found for azoxystrobin MANGANESE LIPOXYGENASE complex one occupying 0 to -0.8 on PC1 and 0.5 to 1 on PC2 and other occupying 0.8 to -1.2 on PC1 and -1 to -1.5 on PC2. The MANGANESE LIPOXYGENASE rosmarinic acid complex showed lowest energy conformations in a energy basin occupying 1.5 to 2.2 on PC1 and 0 to -1 on PC2.

The apo PRE-MRNA-SPLICING FACTOR CEF1 showed a large energy basin in anti-correlating region occupying -0.2 to -3 on PC1 and 1 to -1 on PC2 (**Fig 10D**). In the case of PRE-MRNA-SPLICING FACTOR CEF1 azoxystrobin complex four very small energy basins one in non-correlating region, one in positively correlating region and two in anti-correlating regions were observed. Two relatively large energy basins, compared to apo PRE-MRNA-SPLICING FACTOR CEF1, were found for myricetin PRE-MRNA-SPLICING FACTOR CEF1 complex. Out of these the largest energy basin occupied 0 to -2 on PC1 and 0 to -3 on PC2 and the smaller occupied 0 to -2 on PC1 and 1 to 3 on PC2.

#### 3.5.7. Cluster analysis

**Fig 11:**
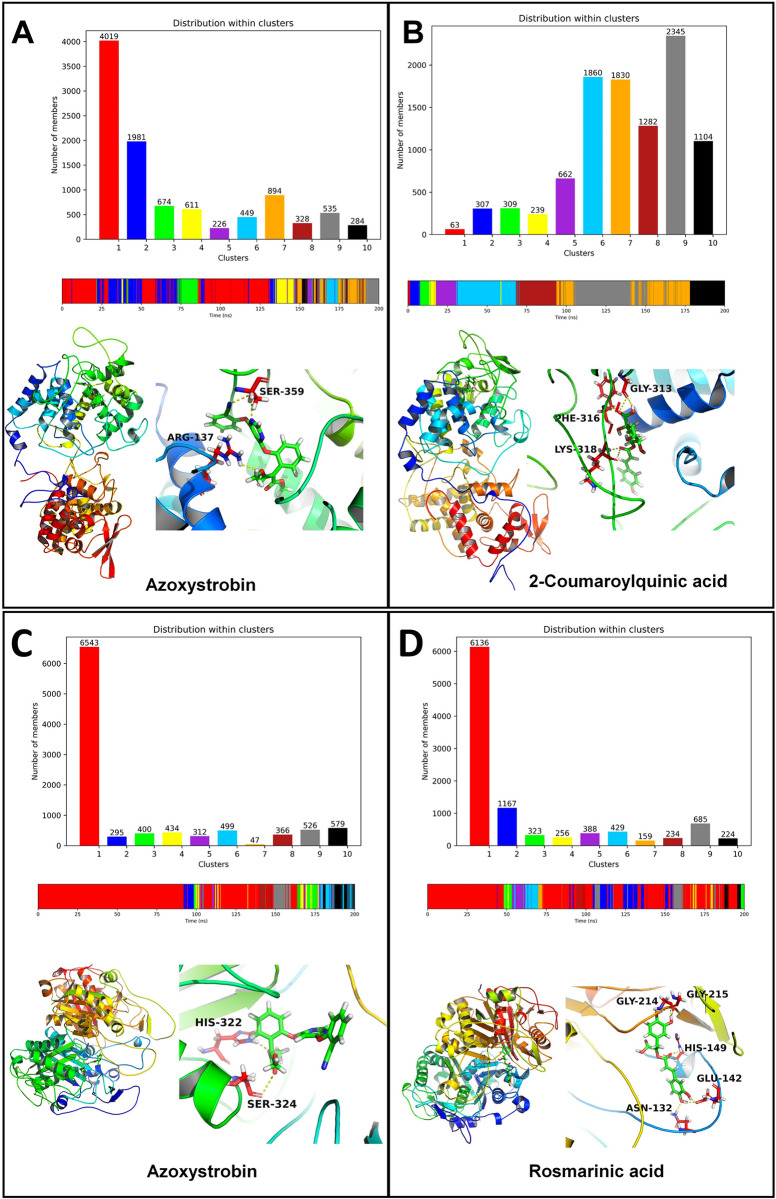
Cluster analysis for CATALASE PEROXIDASES 2 complexes: (A) CATALASE PEROXIDASES 2-azoxystrobin complex and (B) CATALASE PEROXIDASES 2-2-coumaroylquinic acid complex. Cluster analysis for HYBRID PKS-NRPS SYNTHETASE TAS1 complexes: (C) HYBRID PKS-NRPS SYNTHETASE TAS1-azoxystrobin complex and (D) HYBRID PKS-NRPS SYNTHETASE TAS1-rosmarinic acid complex.

The cluster analysis for CATALASE PEROXIDASES 2 azoxystrobin showed that the cluster 1 is the largest cluster. The average structure from this cluster showed the hydrogen bonds between azoxystrobin and Arg137 and Ser359 (**Fig 11A and 11B**). Most of the conformations from this cluster originate between the equilibrated state and around 130 ns. The complex of CATALASE PEROXIDASES 2 with 2-coumaroylquinic acid showed that the cluster 9 is the largest cluster the members of which originate during the simulation period around 100 to 150 ns. The average structure from this cluster showed the hydrogen bonds between 2-coumaroylquinic acid and residues Gly313, Phe316, and Lys318.

The cluster analysis for both the complexes of HYBRID PKS-NRPS SYNTHETASE TAS1 showed that the cluster 1 is the largest cluster with over 6000 conformations in these clusters (**Fig 11C and 11D**). The cluster 1 in the case of HYBRID PKS-NRPS SYNTHETASE TAS1 and azoxystrobin originated from 0 to around 170 ns simulation period, while for complex with rosmarinic acid originated majorly throughout the simulation period. The average structure from the major cluster showed the hydrogen bonds between azoxystrobin and His322 and Ser324 residues. The average structure of cluster for rosmarinic acid HYBRID PKS-NRPS SYNTHETASE TAS1 complex showed hydrogen bonds with Gly214, Gly215, His149, Glu142, and Asn132 residues.

**Fig 12:**
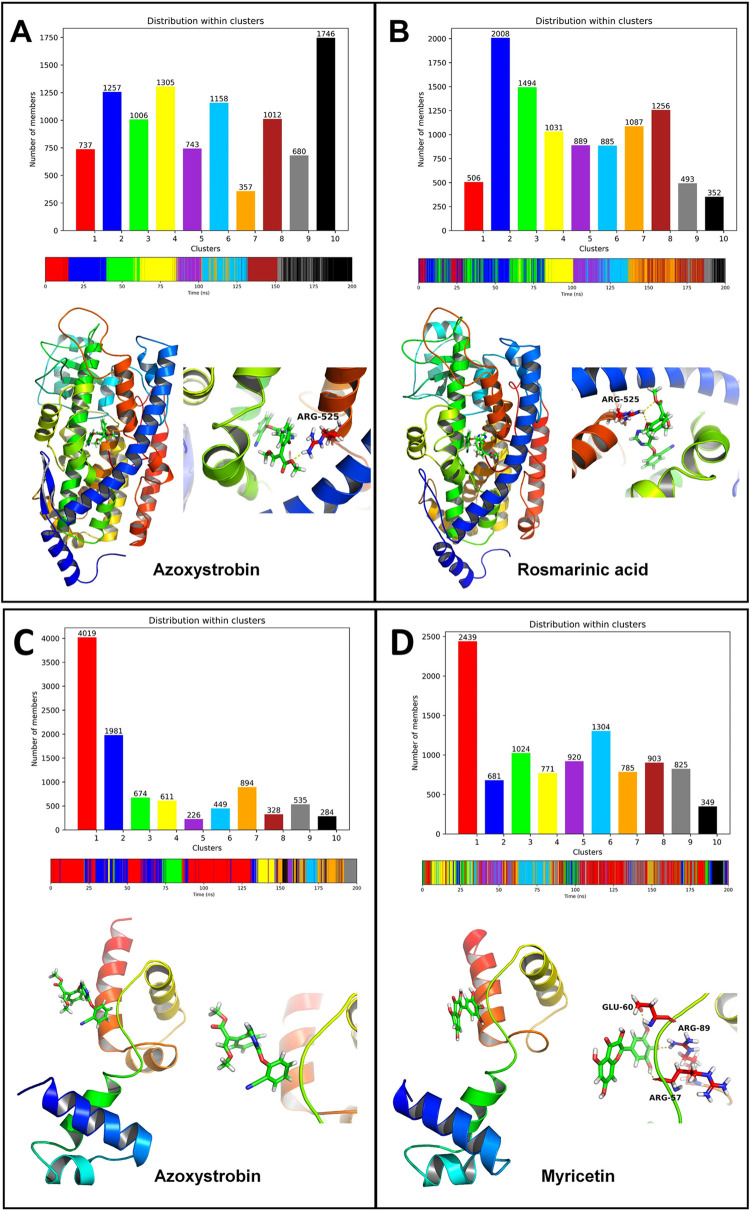
Cluster analysis for MANGANESE LIPOXYGENASE complexes: (A) MANGANESE LIPOXYGENASE-azoxystrobin complex and (B) MANGANESE LIPOXYGENASE-rosmarinic acid complex. Cluster analysis for PMSF complexes: (C) PMSF-azoxystrobin complex and (D) PMSF-myricetin complex.

The cluster analysis for the complex of MANGANESE LIPOXYGENASE with azoxystrobin showed that the cluster 10 is the largest cluster the members of which originated from the simulation period between 150 ns to 200 ns (**Fig 12A and 12B**). The average structure of this cluster showed a hydrogen bond with Arg525. On the other hand for the complex of MANGANESE LIPOXYGENASE with rosmarinic acid showed cluster 2 being the largest cluster originated during the start of simulation to around 80 ns. The average structure from this cluster also showed a hydrogen bond with Arg525.

Cluster 1 was found being the largest cluster for the complexes of PRE-MRNA-SPLICING FACTOR CEF1 with azoxystrobin and myricetin (**Fig 12C and 12D**). Further, the members of this cluster in the case of azoxystrobin PRE-MRNA-SPLICING FACTOR CEF1 complex were found originated during the start of simulation period to around 130 ns. The members of cluster for myricetin PRE-MRNA-SPLICING FACTOR CEF1 complex were found originated during entire simulation period. However, no hydrogen bonds were found in the average structure from the major cluster of PRE-MRNA-SPLICING FACTOR CEF1 azoxystrobin complex, while myricetin PRE-MRNA-SPLICING FACTOR CEF1 complex showed the hydrogen bonds with Glu60, Arg89, and Arg57 residues.

#### 3.5.8. MM-PBSA calculation

The results of MM-PBSA calculations are given in **Table 3**. Overall, azoxystrobin showed lower binding free energies than the other studied ligands such as 2-coumaroylquinic acid and rosmarinic acid, except in the case of PRE-MRNA-SPLICING FACTOR CEF1 complex. In the case of CATALASE PEROXIDASES 2 complexes the binding free energy for azoxystrobin was -102.046 kJ mol^-1^, while the 2-cumaroylquinic acid was -63.156 kJ mol^-1^. In the case of HYBRID PKS-NRPS SYNTHETASE TAS1, azoxystrobin has the binding free energy of -121.849 kJ mol^-1^ and rosmarinic acid has -53.241 kJ mol^-1^. The binding free energy for azoxystrobin in MANGANESE LIPOXYGENASE complex was -93.894 kJ mol^-1^ and that for rosmarinic acid was -66.951 kJ mol^-1^. The binding free energy for azoxystrobin in PRE-MRNA-SPLICING FACTOR CEF1 complex was found higher and unfavorable, while myricetin has lower and favorable binding free energy of -39.999 kJ mol^-1^.

**Table 3:** Results of MM-PBSA calculations.

Complex	van der Waal energy (kJ mol^-1^)	Electrostatic energy (kJ mol^-1^)	Polar solvation energy (kJ mol^-1^)	Solvent accessible surface area energy (kJ mol^-1^)	Binding energy (ΔG_binding_) (kJ mol^-1^)
CATALASE PEROXIDASES 2-Azoxystrobin	-179.464 (1.726)	-6.903 (0.633)	106.053 (2.199)	-21.358 (0.204)	-102.046 (2.688)
CATALASE PEROXIDASES 2-2-Coumaroylquinic acid	-169.215 (1.749)	-15.600 (0.725)	141.378 (1.403)	-19.923 (0.167)	-63.156 (1.793)
HYBRID PKS-NRPS SYNTHETASE TAS1-Azoxystrobin	-168.851 (1.837)	-2.607 (0.435)	69.371 (0.894)	-19.760 (0.161)	-121.849 (1.651)
HYBRID PKS-NRPS SYNTHETASE TAS1-Rosmarinic acid	-113.454 (10.448)	-14.545 (1.495)	89.616 (8.428)	-14.908 (1.162)	-53.241 (7.824)
MANGANESE LIPOXYGENASE-Azoxystrobin	-164.798 (2.136)	-8.674 (0.677)	101.424 (2.462)	-21.905 (0.298)	-93.894 (2.138)
MANGANESE LIPOXYGENASE-Rosmarinic acid	-117.630 (2.733)	-7.953 (1.212)	75.588 (2.810)	-16.963 (0.335)	-66.951 (3.484)
PRE-MRNA-SPLICING FACTOR CEF1 -Azoxystrobin	-15.600 (5.801)	-1.871 (0.923)	31.343 (8.853)	-2.205 (0.762)	11.242 (8.458)
PRE-MRNA-SPLICING FACTOR CEF1 -Myricetin	-107.727 (8.209)	-18.897 (1.691)	98.443 (7.131)	-11.821 (0.612)	-39.999 (5.630)

Standard deviations are given in parentheses

### 3.6. Analysis of fungicide-like properties

The evaluation of fungicide-like properties of the metabolites revealed that quercetin demonstrated high gastrointestinal absorption. Additionally, all the metabolites exhibited no inhibitory effects on CYP2D6. The synthetic accessibility of all the candidates was determined to be higher than 3.20. Among these fungicides, only Myricetin violated the number of hydrogen bond donors required by Lipinski rules, whereas all other medications complied with Lipinski characteristics. According to Lipinski rules, compounds that adhere to these characteristics are considered to be good fungicides [53]. Moreover, solubility was favorable for all metabolites as all the compounds were soluble in water. Furthermore, the molecular weight for all the compounds was less than 400 gm/mol. Overall, the analysis concluded that these metabolites are safe and have the potential to be effective drug candidates (**Table 4**).

**Table 4:** SwissADME properties of top metabolites.

Parameters		Compounds			
		2-Coumaroylquinic acid	Myricetin	Rosmarinic Acid	Quercetin
Physicochemical parameters	Formula	C16H18O8	C15H10O8	C18H16O8	C15H10O7
	Molecular weight gm/mole	338.31	318.24	360.31	302.24
	Num. heavy atoms	24	23	26	22
	Aromatic heavy atoms	6	16	12	16
	Fraction Csp3	0.38	0	0.11	0
	Num. H-bond acceptors	8	8	8	7
	Num. H-bond donors	5	6	5	5
	Molar Refractivity	81.48	80.06	91.4	78.03
	TPSA	144.52	151.59	144.52	131.36
Lipophilicity	Log *P*_o/w_ (iLOGP)	0.88	1.08	1.17	1.63
	Log *P*_o/w_ (XLOGP3)	-0.07	1.18	2.36	1.54
	Log *P*_o/w_ (WLOGP)	-0.46	1.69	1.65	1.99
	Log *P*_o/w_ (MANGANESE LIPOXYGENASEOGP)	-0.54	-1.08	0.9	-0.56
	Log *P*_o/w_ (SILICOS-IT)	-0.15	1.06	1.5	1.54
	Consensus Log *P*_o/w_	-0.07	0.79	1.52	1.23
Pharmacokinetics	GI absorption	Low	Low	Low	High
	BBB permeant	No	No	No	No
	P-gp substrate	No	No	No	No
	CYP1A2 inhibitor	No	Yes	No	Yes
	CYP2C19 inhibitor	No	No	No	No
	CYP2C9 inhibitor	No	No	No	No
	CYP2D6 inhibitor	No	No	No	Yes
	CYP3A4 inhibitor	No	Yes	No	Yes
	Log *K*_p_ (skin permeation) (cm/s)	-8.41	-7.4	-6.82	-7.05
Water solubility	Log *S* (ESOL)	-1.75	-3.01	-3.44	-3.16
	Solubility (mg/Manganese lipoxygenase)	6.04E+00	3.14E-01	1.31E-01	2.11E-01
	Solubility (mol/l)	1.78E-02	9.88E-04	3.63E-04	6.98E-04
	Class	Very soluble	Soluble	Soluble	Soluble
	Log *Sw* (SILICOS-IT)	-0.18	-2.66	-2.17	-3.24
	Solubility (mg/Manganese lipoxygenase)	2.22E+02	6.98E-01	2.41E+00	1.73E-01
	Solubility (mol/l)	6.56E-01	2.19E-03	6.70E-03	5.73E-04
	Class	Soluble	Soluble	Soluble	Soluble
Medicinal chemistry	PAINS	0	1 alert: catechol_A	1 alert: catechol_A	1 alert: catechol_A
	Brenk	1 alert: michael_acceptor_1	1 alert: catechol	2 alerts: catechol, michael_acceptor_1	1 alert: catecho
	Leadlikeness	Yes	Yes	No; 1 violation: MW>350	Yes
	Synthetic accessibility	4.07	3.27	3.38	3.23

### 3.7. Evaluation of compound toxicity

**S5 Table** provides the toxicological assessment of the top metabolites. The LD50 values for these compounds ranged from 1.737 to 2.811 mol/kg, indicating no significant effects on skin sensitivity and acute oral toxicity in rats. Additionally, all of these metabolites showed negative results in terms of AMES toxicity and hepatotoxicity. Furthermore, none of these medications exhibited any adverse effects regarding the inhibition of hERG I.

### 3.8. Evaluation of bioavailability and bioactivity score

During the bioavailability analysis, the majority of our compounds showed proximity to the bioavailability radar, with only one or two characteristics standing out as exceptions (**S6 Fig**). In the bioactivity assay, all four phytocompounds displayed high activity as nuclear receptor ligands and enzyme inhibitors. Notably, 2-Coumaroylquinic acid and Rosmarinic Acid exhibited high activity as GPCR ligands as well. Furthermore, all the compounds demonstrated increased activity across all predicted activities (**S6 Table**).

## 4. Discussion

Rice blast is a highly destructive disease-causing significant crop loss in tropical regions, and fungicide resistance challenges current control options. To address this, exploring natural fungicides becomes crucial due to negative impacts of synthetic fungicides on the environment, humans, and animals [59, 60]. Therefore, advanced bioinformatics methods were used to identify promising phyto-fungicide candidates against rice blast.

In our literature review, we identified four crucial proteins (CATALASE PEROXIDASES 2, HYBRID PKS-NRPS SYNTHETASE TAS1, MANGANESE LIPOXYGENASE, and PRE-MRNA-SPLICING FACTOR CEF1) associated with rice blast infection mechanisms. Furthermore, we compiled a list of 30 plant metabolites with known antifungal properties, showing promise as safe and effective fungicides. Among the reference fungicides, Azoxystrobin and Tricyclazole have been recognized for their efficacy against rice blast. Tricyclazole has demonstrated remarkable effectiveness with a mere 8.41% incidence of leaf blast [61], while azoxystrobin achieves over 73% effectiveness in suppressing rice blast [62]. However, it is important to address the issue of rapidly emerging resistance to both of these fungicides. To tackle this challenge, we employed a comprehensive molecular docking approach to evaluate novel natural fungicide candidates.

Among all ligands, the docking analysis identified 2-Coumaroylquinic acid, Myricetin, Rosmarinic Acid, and Quercetin as the most effective metabolites, surpassing the performance of reference ligands. These metabolites demonstrated higher docking scores and polar binding sites compared to the reference compounds Azoxystrobin and Tricyclazole. Specifically, Rosmarinic Acid showed the highest docking energy for the MANGANESE LIPOXYGENASE and HYBRID PKS-NRPS SYNTHETASE TAS1 proteins, while 2-Coumaroylquinic acid exhibited the best docking score for the CATALASE PEROXIDASES 2 protein. Myricetin demonstrated the highest docking score with the PRE-MRNA-SPLICING FACTOR CEF1 protein. Notably, Myricetin exhibited the highest number of binding polar sites, with eleven identified during the docking step with the CATALASE PEROXIDASES 2 protein. To further investigate the stability and effectiveness of the interactions of these top docked complexes, high throughput molecular dynamics simulation studies were performed.

The RMSD analysis suggested that the CATALASE PEROXIDASES 2 complex with 2-coumaroylquinic acid is comparably less stable as evident from the RMSD reaching beyond 0.6 nm after around 25 ns. While, the standard azoxystrobin stabilizes the CATALASE PEROXIDASES 2 complex quite reasonably compared to 2-coumaroylquinic acid. Although, the RMSD in atoms of 2-coumaroylquinic acid is lower compared to azoxystrobin possibly there is fewer conformational changes in the former ligand necessary to stabilize the CATALASE PEROXIDASES 2. In the case of HYBRID PKS-NRPS SYNTHETASE TAS1 the rosmarinic acid stabilizes the system quite well compared to the standard azoxystrobin. The RMSD around 0.2 nm is almost remained stable throughout the simulation period which suggests the stability of resulting complex. Azoxystrobin on the other hand has slightly larger magnitude of RMSD suggesting slightly lower stabilization of corresponding system. The RMSD in both the ligand atoms remained almost stable after around 50 ns suggesting stable conformations of both the ligands at the binding site. In the case of MANGANESE LIPOXYGENASE complexes both the ligands stabilize the complexes quite well as evident from the lower RMSD of less than 0.2 nm. However, after around 100 ns the complex of rosmarinic acid with MANGANESE LIPOXYGENASE is slightly seen destabilized compared to complex of azoxystrobin which remained stable throughout the simulation. This is further confirmed from the larger magnitude of deviations in RMSD in rosmarinic acid atoms after around 100 ns suggesting larger conformational changes in rosmarinic acid structure. Further the RMSD in azoxystrobin atoms bound to MANGANESE LIPOXYGENASE is slightly larger than the rosmarinic acid suggesting larger deviations in its atoms compared to the starting conformation. The complex of PRE-MRNA-SPLICING FACTOR CEF1 is stabilized quite well with myricetin compared to the azoxystrobin. The RMSD in azoxystrobin bound PRE-MRNA-SPLICING FACTOR CEF1 deviates to very large magnitude after around 125 ns which reach around 1 nm suggesting destabilization of corresponding system. The RMSD in myricetin atoms also suggests minor deviations and corresponding minor conformational changes compared to the azoxystrobin.

The RMSF analysis of CATALASE PEROXIDASES 2 complexes confirms that the overall fluctuations in the side chain atoms of non-terminal residues are minimal for both the ligands suggesting that the bound ligands stabilizing the CATALASE PEROXIDASES 2 complexes. In HYBRID PKS-NRPS SYNTHETASE TAS1 complexes, larger magnitude of fluctuations in the residues around 125 to 150 was evident suggesting binding site flexibility. Further, both the ligands, azoxystrobin and rosmarinic acid produces almost similar magnitude of fluctuations in the HYBRID PKS-NRPS SYNTHETASE TAS1 residues. In the complexes of MANGANESE LIPOXYGENASE both the ligands, azoxystrobin and rosmarinic acid produces lower magnitude of fluctuations in the non-terminal residues. In the case of PRE-MRNA-SPLICING FACTOR CEF1 the RMSF in azoxystrobin bound complex suggests larger conformational changes compared to myricetin bound complex.

The Rg analysis provides the insights of compactness of the systems. In addition to the results of RMSD and RMSF, the results of Rg further corroborate that the CATALASE PEROXIDASES 2 complex with 2-coumaroylquinic acid is less compact compared to azoxystrobin bound CATALASE PEROXIDASES 2 and apo CATALASE PEROXIDASES 2. It is clearly evident that the system with rosmarinic acid bound HYBRID PKS-NRPS SYNTHETASE TAS1 is more compact and thus more stable than the azoxystrobin bound HYBRID PKS-NRPS SYNTHETASE TAS1 and apo HYBRID PKS-NRPS SYNTHETASE TAS1. Both the ligands, azoxystrobin and rosmarinic acid almost equivalently stabilized the MANGANESE LIPOXYGENASE complexes as evident from the almost similar Rg in the complexes of MANGANESE LIPOXYGENASE with these ligands apo MANGANESE LIPOXYGENASE. The PRE-MRNA-SPLICING FACTOR CEF1 Rg for azoxystrobin bound system is slightly lower however deviates to a large magnitude compared to the stable Rg throughout the simulation for myricetin bound system.

The analysis of SASA further confirmed that the CATALASE PEROXIDASES 2 bound to 2-coumaroylquinic acid has larger SASA suggesting larger parts of CATALASE PEROXIDASES 2 exposed to solvent or cavity formation. In HYBRID PKS-NRPS SYNTHETASE TAS1 and MANGANESE LIPOXYGENASE the rosmarinic acid complexes showed lower SASA which is suggestive of better stability and lesser regions of these proteins exposed to solvent. In the case of PRE-MRNA-SPLICING FACTOR CEF1 myricetin showed lesser SASA and thus probably more stable complex is evident.

The hydrogen bond analysis suggested that in the CATALASE PEROXIDASES 2, HYBRID PKS-NRPS SYNTHETASE TAS1 and MANGANESE LIPOXYGENASE complexes azoxystrobin had fewer numbers of hydrogen bonds than the ligands 2-coumaroylquinic acid and rosmarinic acid. More the number of hydrogen bond better could be the binding affinity and consequent stability of the system. However, in PRE-MRNA-SPLICING FACTOR CEF1 neither azoxystrobin nor myricetin could form consistent hydrogen bonds.

The contact analysis revealed that 2-coumaroulquinic acid bound to CATALASE PEROXIDASES 2 produces consistent and more frequent contacts with the binding site residues Phe316, Lys318, Gly313, His314, and Gly317 compared to azoxystrobin which could make such consistent and frequent contact with His141 only. This suggests that 2-coumaroylquinic acid may have more favorable binding interactions. Similarly, in the case of HYBRID PKS-NRPS SYNTHETASE TAS1, rosmarinic acid produced consistent contacts with His149, Gly215, and Gly214, whereas azoxystrobin could not form any consistent contact. This suggests the more favorable binding affinity of rosmarinic acid compared to azoxystrobin in HYBRID PKS-NRPS SYNTHETASE TAS1. In MANGANESE LIPOXYGENASE complexes both the ligands produced consistent contacts with Arg525 and both the ligands, azoxystrobin and rosmarinic acid, may have similar binding affinities. In the case of PRE-MRNA-SPLICING FACTOR CEF1 myricetin having substantial and more numbers of contacts, compared to very weak contacts with azoxystrobin, may have the more favorable binding affinity. The trajectories isolated at different time intervals further confirmed these results and suggest that the ligands 2-coumaroylquinic acid, rosmarinic acid, and myricetin may have better binding affinities compared to the standard azoxystrobin.

Gibb’s energy analysis revealed that the 2-coumaroylquinic acid give rise to large number of stable conformations of CATALASE PEROXIDASES 2 complex in lowest energy basins in CATALASE PEROXIDASES 2. While the azoxystrobin showed fewer and smaller number of conformations existing in low energy basins. Similarly, rosmarinic acid produced large number of stable conformations compared to azoxystrobin in the case of HYBRID PKS-NRPS SYNTHETASE TAS1 and MANGANESE LIPOXYGENASE complexes. Myricetin is found to stabilize and large numbers of low energy conformations were seen in larger low energy basins. Overall, the Gibb’s energy analysis conformed that the ligands 2-coumaroylquinic acid, rosmarinic acid and myricetin produces the more favorable and lowest energy conformations of each corresponding proteins under study, compared to azoxystrobin.

The cluster analysis further corroborates the results of contact frequency analysis and Gibb’s free energy analysis where the major cluster exists for 2-coumaroylquinic acid in complex with CATALASE PEROXIDASES 2 having the key hydrogen bond interactions in the average structure with residues Gly313, Phe316, and Lys318. The major cluster for the azoxystrobin on the other hand showed fewer interactions in the average structure of the cluster, which further confirms more evident favorable affinity of 2-coumaroylquinic acid with CATALASE PEROXIDASES 2. Similarly, rosmarinic acid also has for number of key interactions as seen in the major cluster than the azoxystrobin in the case of HYBRID PKS-NRPS SYNTHETASE TAS1. Here, the hydrogen bond interactions of rosmarinic acid with Gly215, Gly214, His149, Glu142, and Asn132 were also confirmed as seen in the contact frequency analysis. Further, the results of cluster analysis were corroborate with the major clusters for azoxystrobin and rosmarinic acid complexes with MANGANESE LIPOXYGENASE, where the average structures from the clusters showed the key interaction with Arg525. In the case of PRE-MRNA-SPLICING FACTOR CEF1 the major cluster of myricetin showed the interactions with Glu60, Arg89, and Arg57, while no interactions were evident in the major cluster of azoxystrobin, which further confirms the favorable binding affinity of myricetin.

The MM-PBSA analysis for CATALASE PEROXIDASES 2 complexes suggested that due to lower van der Waal energy and lower polar salvation energy the binding free energy for azoxystrobin is better than 2-coumaroylquinic acid. Similarly, in the case of HYBRID PKS-NRPS SYNTHETASE TAS1 and MANGANESE LIPOXYGENASE complexes the influence of van der Waal energy and polar salvation energy results in the more favorable binding affinity of azoxystrobin than the rosmarinic acid. In the case of PRE-MRNA-SPLICING FACTOR CEF1 myricetin is clearly has more favorable binding affinity than the azoxystrobin due to lower van der Waal energy. The MM-PBSA analysis is just indicative of binding free energies in absence of enthalpic contributions.

The majority of the metabolites exhibited Lipinski characteristics, with the exception of Myricetin, which only violated the Lipinski rule in terms of the number of hydrogen bond donors [63]. However, all other metabolites adhered to the Lipinski rule, as they had a molecular weight below 500 Dalton, fewer than 5 hydrogen bond donors, fewer than 10 hydrogen bond acceptors, and a logP value below 5 (**Table 4**). These results suggest that the selected natural metabolites have the potential to penetrate the fungal cell membrane and act as fungicides. Previous research has shown that compounds following the Lipinski rule of five are considered safe and effective fungicides for plants [53]. Our top metabolites also meet these criteria for effective fungicides. Moreover, all the metabolites displayed good solubility, further confirming their efficacy.

In terms of safety, toxicity analysis revealed that none of the top metabolites caused skin sensitivity or toxicity (**S5 Table**) indicating their non-harmful nature. Overall, the toxicity analysis concluded that these metabolites are safe for the environment, humans, and other animals. A bioactivity assay was conducted to assess the durability of the top metabolites as ligands. All four natural metabolites exhibited increasing activity as nuclear receptor ligands and enzyme inhibitors. In the comprehensive analysis, each of these metabolites demonstrated biological activity, with a score value greater than -0.50. These fungicides could be used as potential natural fungicide using different linker, adapters and excipients in fungicide formulation and opens a big door in *in vivo* and *in vitro* analysis for confirming the evaluations.

## 5. Conclusion

The study highlights the significance of using natural plant metabolites as potential fungicides to combat rice blast. The findings demonstrate the effectiveness and safety of these compounds, offering sustainable and eco-friendly alternatives to synthetic fungicides. The identification of novel phytocompounds with high binding affinities opens up promising prospects for practical applications in agriculture. Implementing these potential natural fungicides in the future could significantly improve rice blast management, ensuring better crop yields, reduced environmental impact, and enhanced agricultural sustainability.

## Supporting information

S1 Fig 3D visualization of target proteins A) CP2, B) HPNST, C) ML, D) PMSFC and Predicted active site of target proteins E) CP2, F) HPNST, G) ML, H) PMSFC.
(DOCX)

S2 Fig Contact frequency analysis for CP2 complexes. A) CP2-azoxystrobin complex and B) CP2- 2-coumaroylquinic acid complex. (The left side panel shows the contact plot based on mean smallest distance between residues, where the last residue on both the axis is respective ligand. The right side panel shows the contact frequency plot for residues within 3.5 Å from respective ligand).
(DOCX)

S3 Fig Contact frequency analysis for HPNST complexes. A) HPNST-azoxystrobin complex and B) HPNST-rosmarinic acid.
(DOCX)

S4 Fig Contact frequency analysis for ML complexes. A) ML-azoxystrobin complex and B) ML-rosmarinic acid.
(DOCX)

S5 Fig Contact frequency analysis for PMSFC complexes. A) PMSFC-azoxystrobin complex and B) PMSFC-rosmarinic acid.
(DOCX)

S6 Fig Bioavailability radar of top metabolites A) 2-Coumaroylquinic acid, B) Myricetin, C) Quercetin and D) Rosmarinic Acid.
(DOCX)

S1 Table Key functions of target proteins. (DOCX)

S2 Table Predicted active site of the target proteins. (DOCX)

S3 Table Enlistment of antifungal plant metabolites. (DOCX)

S4 Table Docking result of target protein against metabolites. (DOCX)

S5 Table Toxicity parameter of selected metabolites. (DOCX)

S6 Table Bioactivity assay prediction of top metabolites. (DOCX)

## Abbreviations

GDP: gross domestic product
SDF: Structure data file
PDB: Protein Data Bank
RCSB: Research Collaboratory for Structural Bioinformatics
PYMOL: proprietary molecular visualization system
pkCSM: predicting small-molecule pharmacokinetic and toxicity properties using graph-based signatures
LD 50: Lethal Dose 50
GI: Gestrointestinal
TPSA: Topological Polar Surface Area
ICM: Ion channel modulator
KI: Kinase Inhibitor
NRL: Nuclear receptor ligand
PI: Protease inhibitor
EI: Enzyme inhibitor
CASTp: Computed Atlas of Surface Topography of proteins
