# Supplementary material for: Antifungal plant flavonoids identified in silico with potential to control rice blast disease caused by Magnaporthe oryzae
Source: PLoS One. 2024 Apr 5;19(4):e0301519. doi: 10.1371/journal.pone.0301519 (PMC10997076; doi:10.1371/journal.pone.0301519)
Supplement: S1 Fig — 3D visualization of target proteins A) CP2, B) HPNST, C) ML, D) PMSFC and Predicted active site of target proteins E) CP2, F) HPNST, G) ML, H) PMSFC. (DOCX) [file pone.0301519.s001.docx]

**
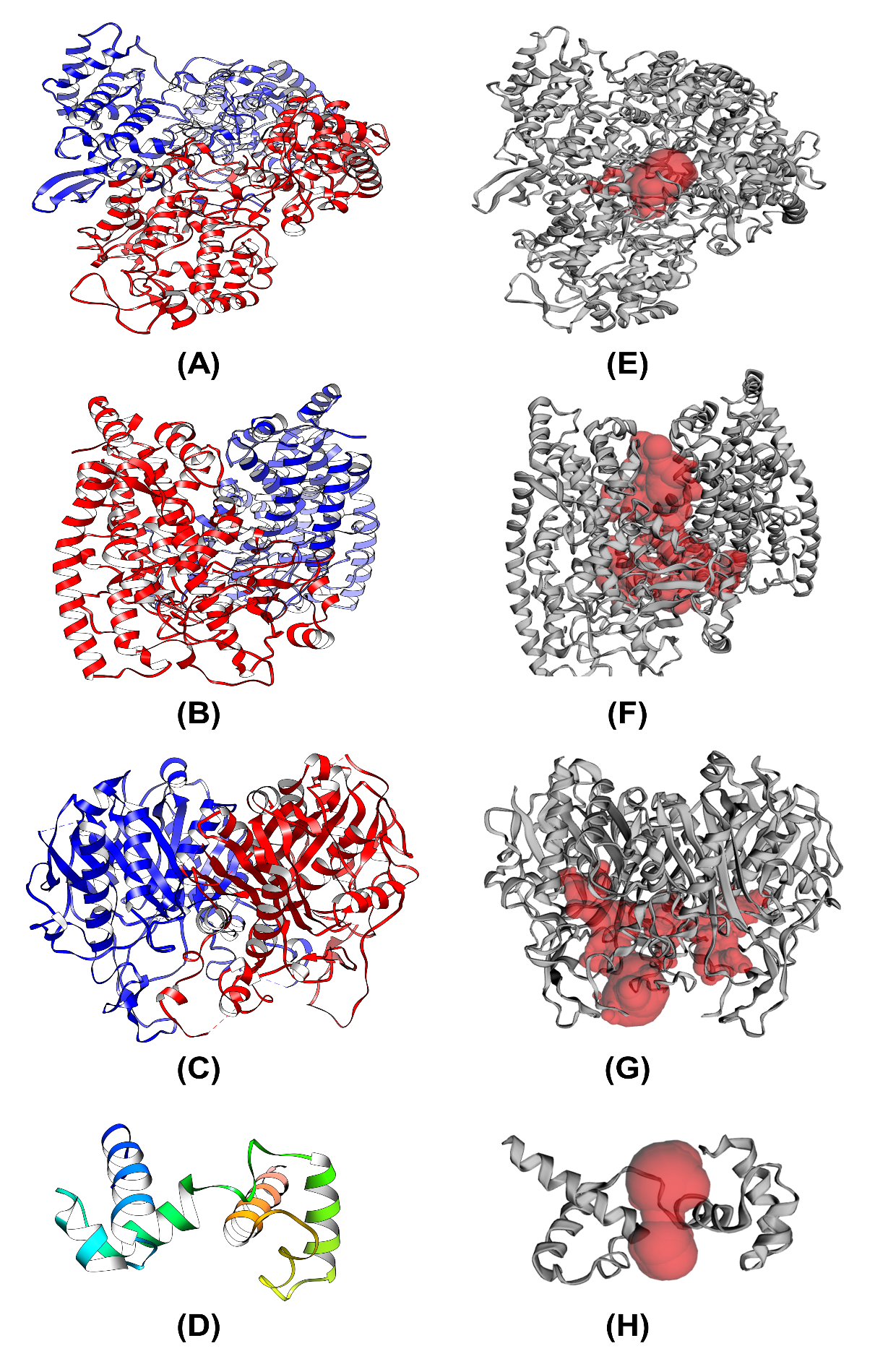
**

**S1 Figure:** 3D visualization of target proteins A) CP2, B) HPNST, C) ML, D) PMSFC and Predicted active site of target proteins E) CP2, F) HPNST, G) ML, H) PMSFC
