# Supplementary material for: Antifungal plant flavonoids identified in silico with potential to control rice blast disease caused by Magnaporthe oryzae
Source: PLoS One. 2024 Apr 5;19(4):e0301519. doi: 10.1371/journal.pone.0301519 (PMC10997076; doi:10.1371/journal.pone.0301519)
Supplement: S2 Fig — A) CP2-azoxystrobin complex and B) CP2- 2-coumaroylquinic acid complex. (The left side panel shows the contact plot based on mean smallest distance between residues, where the last residue on both the axis is respective ligand. The right side panel shows the contact frequency plot for residues within 3.5 Å from respective ligand). (DOCX) [file pone.0301519.s002.docx]

**
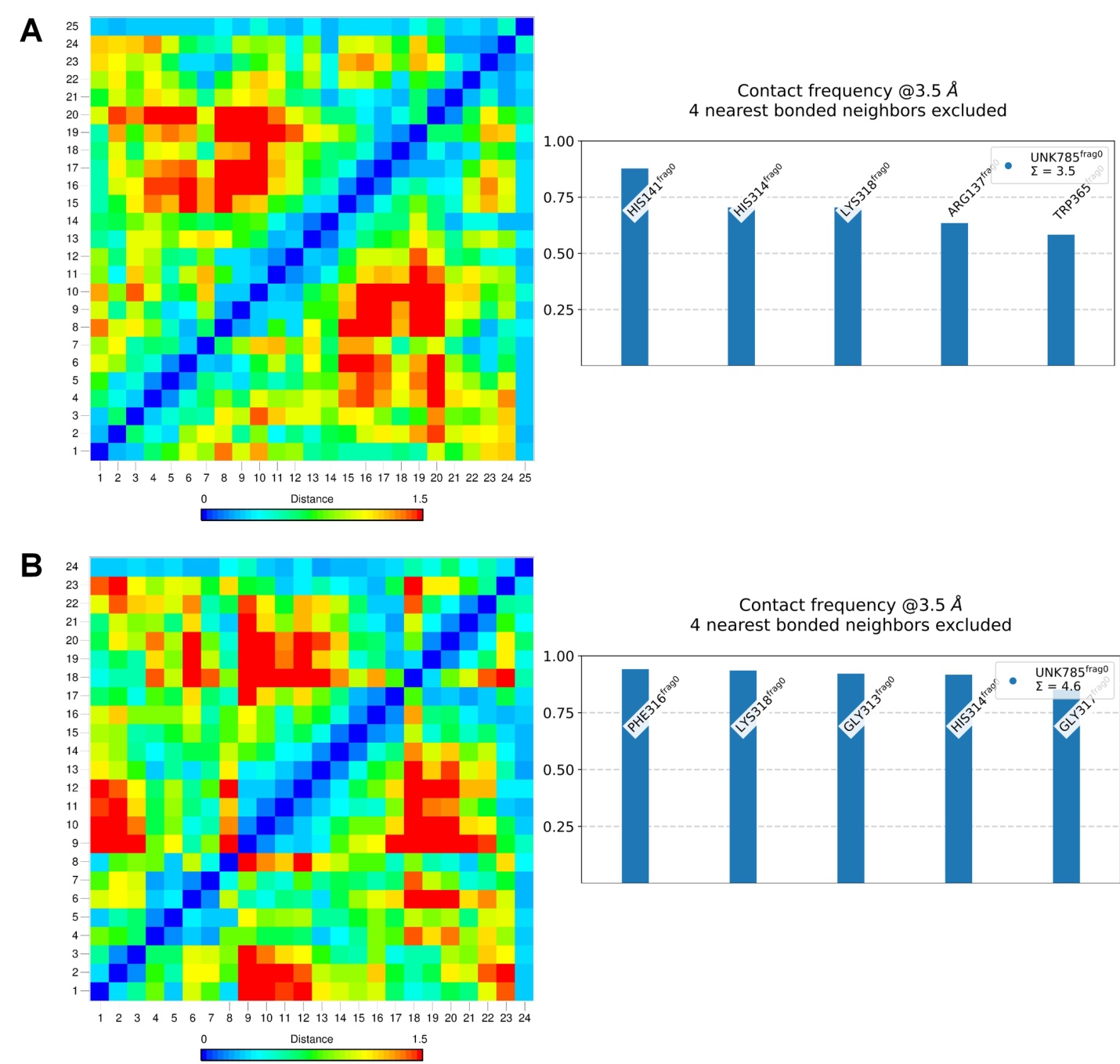
**

**S2 Figure:** Contact frequency analysis for CP2 complexes. A) CP2-azoxystrobin complex and B) CP2- 2-coumaroylquinic acid complex. (The left side panel shows the contact plot based on mean smallest distance between residues, where the last residue on both the axis is respective ligand. The right side panel shows the contact frequency plot for residues within 3.5 Å from respective ligand.)
