# Supplementary material for: Antifungal plant flavonoids identified in silico with potential to control rice blast disease caused by Magnaporthe oryzae
Source: PLoS One. 2024 Apr 5;19(4):e0301519. doi: 10.1371/journal.pone.0301519 (PMC10997076; doi:10.1371/journal.pone.0301519)
Supplement: S4 Fig — A) ML-azoxystrobin complex and B) ML-rosmarinic acid. (DOCX) [file pone.0301519.s004.docx]

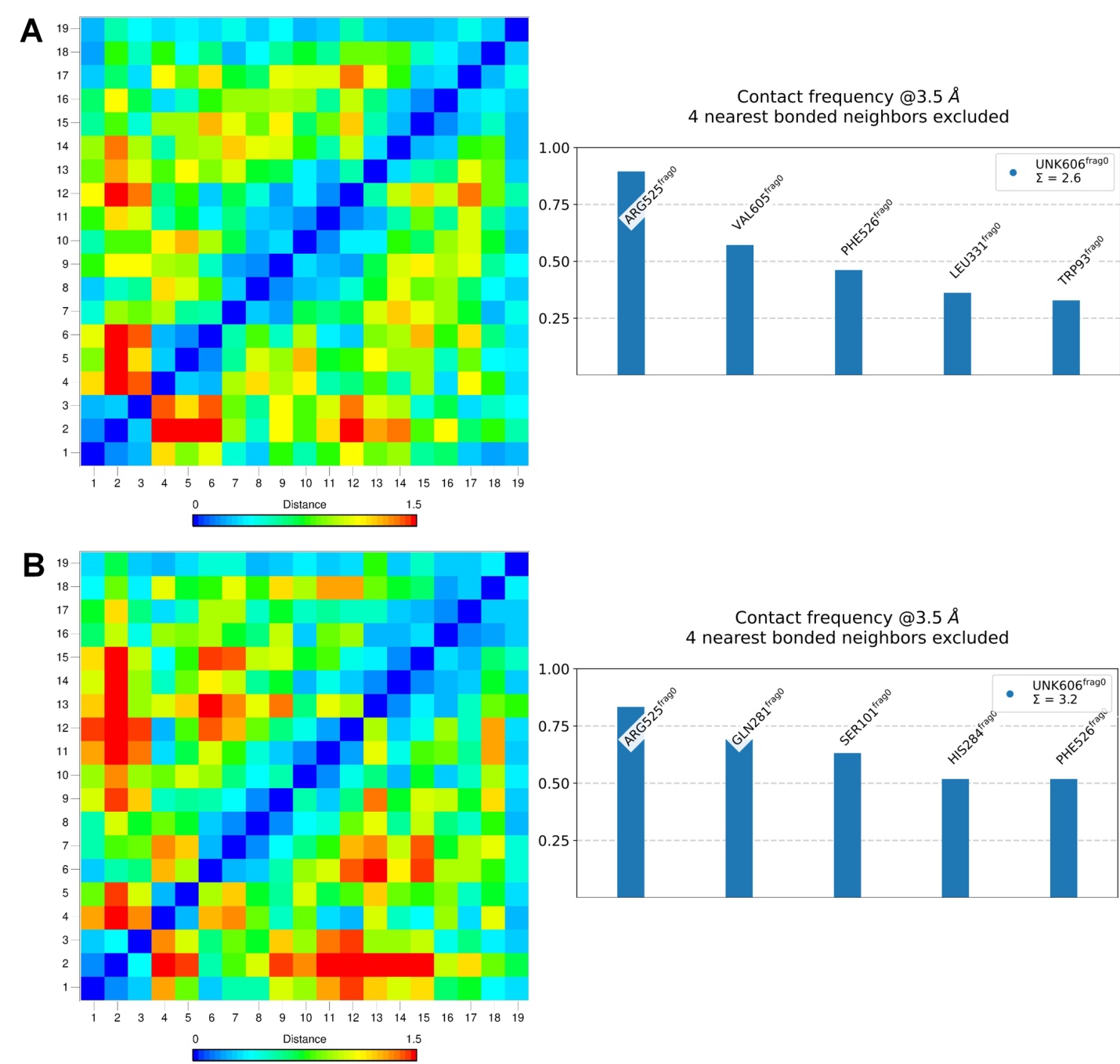


**S4 Figure:** Contact frequency analysis for ML complexes. A) ML-azoxystrobin complex and B) ML-rosmarinic acid
