# Supplementary material for: Antifungal plant flavonoids identified in silico with potential to control rice blast disease caused by Magnaporthe oryzae
Source: PLoS One. 2024 Apr 5;19(4):e0301519. doi: 10.1371/journal.pone.0301519 (PMC10997076; doi:10.1371/journal.pone.0301519)
Supplement: S6 Fig — Bioavailability radar of top metabolites A) 2-Coumaroylquinic acid, B) Myricetin, C) Quercetin and D) Rosmarinic Acid. (DOCX) [file pone.0301519.s006.docx]

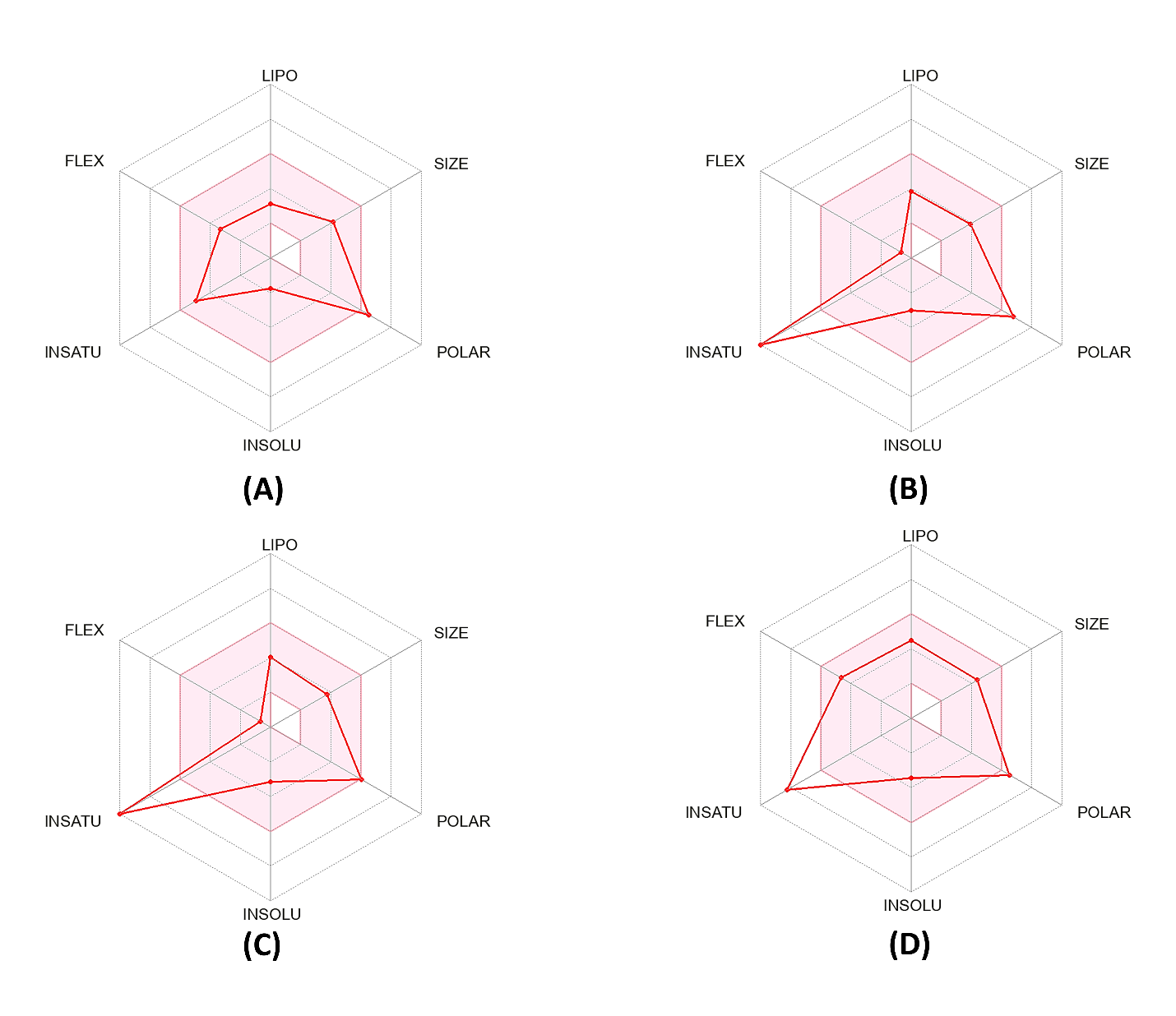


**S6 Figure:** Bioavailability radar of top metabolites A) 2-Coumaroylquinic acid, B) Myricetin, C) Quercetin and D) Rosmarinic Acid.
