# Supplementary material for: Antifungal plant flavonoids identified in silico with potential to control rice blast disease caused by Magnaporthe oryzae
Source: PLoS One. 2024 Apr 5;19(4):e0301519. doi: 10.1371/journal.pone.0301519 (PMC10997076; doi:10.1371/journal.pone.0301519)
Supplement: S1 Table — (DOCX) [file pone.0301519.s007.docx]

**S1 Table:** Key functions of target proteins

| **Protein** | **PDB ID** | **Functions** |
| --- | --- | --- |
| Catalase-peroxidases 2 (CP2) | 3UT2 | The enzyme Catalase peroxidase 2 contributes to the fungal defense against H2O2 accumulated in rice epidermal cells during the early infection stage. Enzyme with broad-spectrum peroxidase activity and catalase activity which gives rise to H2O2 resistance in hyphae. Early in the plant infection process, play an antioxidative role in fungal defense against the host's production of H2O2 (oxidative burst) [1]. Hydrogen peroxide is converted by catalase into water and triplets oxygen [2]. Inhibiting this protein can make it difficult for the pathogen to survive if infection arises |
| Hybrid PKS-NRPS synthetase TAS1 (HPNST) | 6KOG | The PKS-NRPS synthetase Hybrid TAS1 is a member of the gene cluster that regulates the manufacture of the toxin tenuazonic acid (TeA), which inhibits protein biosynthesis on ribosomes by reducing protein release and an inhibitor of photosynthetic processes [3]. By blocking this protein, these mechanisms that help the pathogen to cause rice blast disease could be narrowed.  TAS1 is a unique NRPS-PKS hybrid enzyme that begins with a C-A-PCP module [4]. In contrast to other NRPS/PKS hybrid enzymes, TAS1's PKS part has just a ketosynthase (KS) domain, which is required for TAS1 activity. This KS domain is classified as a separate clade adjacent to the type I PKS KS domain based on phylogenetic study. The TAS1 KS domain completes the final cyclization step for the release of tenuazonic acid [5] |
| Manganese lipoxygenase (ML) | 5FNO | When *Magnaporthe oryzae* (Mo) causes rice blast disease, It expresses manganese lipoxygenase complex (Mo-MnLOX) which plays a vital role in the infection process. The manganese lipoxygenase has great biological importance in rice blast disease [6]. It initiates in secretion of enzyme MnLOX that promotes plant necrosis, invasive hyphal development, and infection. n of α-linolenic acid [7]. |
| Pre-mRNA-splicing factor CEF1(PMSFC) | 6JUI | Pre-mRNA splicing factor competes with hnRNP A1 to increase the use of proximal alternative 5' splice sites and possesses an activity necessary for general splicing in vitro. It controls the cell cycle and pre-mRNA splicing [8]. |

**References:**

[1] Tanabe, S., et al., *The role of catalase-peroxidase secreted by Magnaporthe oryzae during early infection of rice cells.* Molecular Plant-Microbe Interactions. **24**(2) 2011, p. 163-171.

[2] Trivedi, A., et al., *Chapter 4 - Redox Biology of Tuberculosis Pathogenesis*, in *Advances in Microbial Physiology*, R.K. Poole, Editor. Academic Press. 2012, p. 263-324.

[3] Chen, S. and S. Qiang, *Recent advances in tenuazonic acid as a potential herbicide.* Pesticide biochemistry and physiology. **143**2017, p. 252-257.

[4] Yun, C.-S., et al., *Biosynthesis of the mycotoxin tenuazonic acid by a fungal NRPS–PKS hybrid enzyme.* Nature communications. **6**(1) 2015, p. 8758.

[5] Hou, Y., et al., *Comparative genomics of pathogens causing brown spot disease of tobacco: Alternaria longipes and Alternaria alternata.* PloS one. **11**(5) 2016, p. e0155258.

[6] Wennman, A., et al., *Crystal Structure of Manganese Lipoxygenase of the Rice Blast Fungus Magnaporthe oryzae.* J Biol Chem. **291**(15) 2016, p. 8130-9.

[7] Oliw, E.H., *Iron and manganese lipoxygenases of plant pathogenic fungi and their role in biosynthesis of jasmonates.* Archives of biochemistry and biophysics. **722**2022, p. 109169.

[8] Galagan, J.E., et al., *The genome sequence of the filamentous fungus Neurospora crassa.* Nature. **422**(6934) 2003, p. 859-68.
