# Supplementary material for: Antifungal plant flavonoids identified in silico with potential to control rice blast disease caused by Magnaporthe oryzae
Source: PLoS One. 2024 Apr 5;19(4):e0301519. doi: 10.1371/journal.pone.0301519 (PMC10997076; doi:10.1371/journal.pone.0301519)
Supplement: S2 Table — (DOCX) [file pone.0301519.s008.docx]

**S2 Table:** Predicted active site of the target proteins

| **Protein** | **Area (SA) Å^2^** | **Volume (SA) Å^3^** | **Predicted Active sites** |
| --- | --- | --- | --- |
| CP2 | 979.960 | 922.351 | VAL80, LEU81, GLN83, PHE84, GLN85, PRO86, GLN88, ASN89, PRO90, LEU91, GLY93, PHE95, ASP96, TYR 97, ALA98, THR145, ARG152, GLY153, GLY154, GLY156, MET157, GLN159, ARG161, ALA163, PRO164, LEU165, LYS185, ASN190, SER193, TRP194,GLY219, ARG220, ALA221, ASP222, THR223, TRP224, GLN225, ASP227, ALA229, VAL230, TRP232, ASP462, LEU463, THR467, ARG468, LEU470, TYR527, ASN529, SER530, LYS532, SER657, GLN658, THR660, LEU661, THR662, LYS773, ASN776, LEU777, ARG779, PHE780, LEU782, LYS783, VAL784, LEU78, ARG82, PRO663, GLU746, ALA749, ILE750, GLU752, VAL753, GLU756 |
| HPNST | 2184.725 | 1396.042 | PHE40, LEU46, GLY47, PHE48, ALA49, TYR60, MET62, ASP87, GLN87, LYS88, ALA121, THR123, ASN124, THR125, LEU127, SER128,ARG130, PHE146, HIS149, TYR150, GLN152, LEU153, ASP155, LEU154, CYS156, PRO157, ILE158, THR174, LEU175, ASN176, ALA178, CYS179, SER207, MET208, ALA209, TYR210, PRO211, GLN212, GLU213, GLY214, GLY215, TYR216, SER225, SER226, VAL228, PHE229, CYS247, PRO247, ALA248, ASP249, ASP281, LYS286, LEU289, GLY290, VAL291, PRO292, GLN297, HIS322, GLY323, SER324, THR326, TRP328, GLY329, ASP330, GLU333, ASN376, GLU378, ALA379, LEU438, GLY439, TYR440, GLY441, 442, SER443, ASN444, PHE40, TYR60, MET62, ASP85, GLN87, LYS88, ALA121, THR123, ASN124, THR125, LEU127, SER128, ASN132, GLY134, SER135, GLU141, GLU142, GLN144, PHE146, ASN148, HIS149, TYR150, GLN151, GLN152, LEU153, ASP155, CYS156, PRO157, ILE158, THR174, LEU175, ASN176, ALA178, CYS179, SER207, MET208, ALA209, TYR210, PRO211, GLN212, GLU213, GLY214, GLY215, TYR216, SER226, VAL228, PHE229, CYS245, PRO247, ALA248, ASP249, ASP281, LYS286, LEU289, GLY290, VAL291, PRO292, GLN297, HIS322, GLY323, SER324, THR326, TRP328, GLY329, ASP330, GLU333, ASN376, GLU378, ALA379, LEU438, GLY439, TYR440, GLY441, GLY442, SER443, ASN444, |
| ML | 3294.823 | 2471.753 | LEU155, MET159, ARG171, HIS173, PRO174, GLU176, THR192, LYS193, THR194, ALA198, LEU199, ALA201, ARG204, LEU233, PRO235, ARG236, SER237, ASN238, GLN239, PHE240, ARG364, TYR367, LEU368, ASP369, THR370, ARG373, ALA374, GLY376, LEU377, VAL378, ASP379, ALA380, ASP381, TYR382, GLY383, PRO384, GLU385, LEU386, PRO387, HIS388, PHE389, TYR392, GLU393, ASP394, SER396, ARG397, VAL399, GLU400, ARG403, ARG407, VAL410, ASP411, ASP418, GLU444, PRO445, THR450, ARG451, GLU452, ARG453, VAL455, GLU456, THR459, HIS460, TRP463, ARG568, ASP471, GLU572, ALA575, ARG576, ARG577, PHE578, ASP579, ARG581, LEU583, SER584, GLN585, GLY586, LEU155, MET159, ARG171, PRO174, ALA201, ALA202, ARG204, LEU233, PRO235, ARG236, SER237, ASN238, GLN239, PHE240, ARG364, TYR367, LEU368, ASP369, THR370, ARG373, LEU377, VAL378, ASP379, ALA380, ASP381, TYR382, GLY383, PRO384, GLU385, LEU386, PRO387, HIS388, PHE389, TYR392, GLU393, ASP394, SER396, ARG397, VAL399, GLU400, VAL401, ARG403, ARG404, PHE405, ARG407, SER408, VAL410, ASP411, GLU416, ASP418, GLU444, PRO445, GLY446, PRO447, ASP449, THR450, ARG451, GLU452, ARG453, VAL455, GLU456, THR459, HIS460, TRP463, ALA558, ARG561, PHE562, ARG564, ALA565, ARG568, ASP571, GLU572, ALA575, ARG576, ARG577, PHE578, ASP579, ASP580, ARG581, LEU583, SER584, GLN285, GLY586, |
| PMSFC | 176.379 | 255.012 | GLY7, VAL8, TRP9, LYS42, GLN43, LYS45, ALA46, ARG47, ASN49, GLU50, TRP51, ARG81, THR90, ALA91, ASN92, GLN93, GLU96 |
