# Supplementary material for: Antifungal plant flavonoids identified in silico with potential to control rice blast disease caused by Magnaporthe oryzae
Source: PLoS One. 2024 Apr 5;19(4):e0301519. doi: 10.1371/journal.pone.0301519 (PMC10997076; doi:10.1371/journal.pone.0301519)
Supplement: S3 Table — (DOCX) [file pone.0301519.s009.docx]

**S3 Table:** Enlistment of antifungal plant metabolites

| **Sl No.** | **Metaboli Tes** | **Class** | **Pubc Hem Cid** | **Source** | **Biological Effects** | **References** |
| --- | --- | --- | --- | --- | --- | --- |
|  | Myricetin | Hexahydroxyflavone | 5281672 | *Rosa Damascena* | Antifungal Activity, Cyclooxygenase 1 Inhibitor, Antineoplastic Agent, Antioxidant, Plant Metabolite, Food Component, A Hypoglycemic Agent And A Geroprotector, Strong Anti-Oxidant, Anticancer, Antidiabetic And Anti-Inflammatory Activities | [9] |
|  | Chamazulene | Sesquiterpenes | 10719 | *A. Millefolium* | Antifungal Activity, Antiseptic, Antibacterial,  Anti-Inflammatory  Antiallergic, | [10] |
|  | Geraniol | Monoterpenoid | 637566 | *Pelargonium Graveolens* | Antifungal Activity, Inhibited Mycelial Growth, Antibacterial, Synergistic Effect, Antioxidant, Anti-Inflammatory And Anticancer Agent | [11] |
|  | Linalool | Acyclic Monoterpenoid. | 6549 | *Stachys Inflata Benth* | Antifungal Activity, Antimicrobial Agent | [12] |
|  | 1-Methylxanthine | Xanthines | 80220 | *Theobroma Cacao* | Antifungal Activity | [13] |
|  | Emodin | Aromatic Polyketide. | 3220 | *Cassia Alata* | Antifungal , Diuretic, Antibacterial, Antiulcer, Anti-Inflammatory, Anticancer, Inhibit Bacterial Growth | [14] |
|  | Camphor | Terpenoid Ketones | 2537 | *Artemisia Sieberi Besser* | Antifungal Activity, Antibacterial Activity | [15] |
|  | Bornyl Acetate | Bicyclic Monoterpenoids | 6448 | *Salvia Eremophila Boiss.* | Antifungal Activity, Antimicrobial Activity, Potent Chemoattractant Agent, Food Additive, Flavoring Agent and Odor Agent. | [16] |
|  | Glaucine | Alkaloids | 16754 | *M. Canis (Mc)* | Antifungal Activity, Laxative, Hypnotic, Antidiabetic Agent, Antimicrobial Agents | [17] |
|  | Piperitenone Oxide | Oxepanes | 442497 | *Mentha Spicata* | Antifungal Activity, Antimicrobial Agents | [18] |
|  | Sparteine | Alkaloids | 644020 | *Retama Monosperma* | Antifungal Activity, Anti-Inflammatory, Anti-Convulsant and Anti-Cancer | [19] |
|  | Thymol | Phenols And A Monoterpenoid. | 6989 | *Satureja Hortensis* | Antifungal Activity, Antibacterial, Antioxidant, Antibiotics and Antimycotics | [20] |
|  | Carvacrol | Aromatic Monoterpenoids | 10364 | *Satureja Hortensis* | Antifungal Activity, Anti-Infective Agents and Anti-Virulence | [21] |
|  | Canavanine | Non-Proteinogenic L-Alpha-Amino Acid | 439202 | *Canavalia Species.* | Antifungal activity, Antibacterial activity, Effects Regulatory and Catalytic Reactions Of Arginine Metabolism, Arginine Uptake, Formation Of Structural Components, and Other Cellular Processes, Can Disrupt Critical Reactions Of RNA And DNA Metabolism As Well As Protein, Antiviral | [22] |
|  | Jasmonic Acid | Isoprenoids | 5281166 | *Jasminum Grandiflorum* | Antifungal Activity, Coordinate Responses to Biotic and Abiotic Threats, Naturally Occurring Growth Regulator, Anti-Cancer, Anti-Inflammatory, Antioxidant, | [23] |
|  | Serotonin | Amino Compound | 5202 | *Mucuna Pruriens* | Antifungal Activity, Antimicrobial, Anticancer, And HIV-1 Integrase Inhibitory Activities, Anti-Inflammatory, | [24] |
|  | Allicin | Thiosulfinate | 65036 | *Allium Sativum* | Antifungal Activity, Anticancer, Anti-Inflammatory, Antimicrobial, Antioxidant, Cardio protective and Immunomodulatory | [25] |
|  | Rosmarinic Acid | Hydroxylated Compound | 5281792 | *Hymenocrater Calycinus (Boiss.) Benth.* | Antifungal Activity, Anti-Inflammatory, Antimicrobial and Antioxidant | [26] |
| 19. | 2-Coumaroyl Quinic Acid | Organic Compounds | 6441280 | *Eucalyptus Globulus* | Antifungal Activity and Antibacterial Activity | [27] |
| 20. | Catechol | Benzenediol | 289 | *Nicotiana Attenuata* | Antifungal Activity , Antioxidant and Fungicide | [28] |
| 21. | Sinapaldehyde | Cinnamaldehydes (Organic Compound) | 5280802 | *Stereospermum Colais* | Antifungal Activity, Antibacterial and Antioxidant | [29] |
| 22. | Protoemetine | Alkaloids | 443421 | *Alangium Salviifolium* | Antifungal, Antibacterial, Antiviral .And Cytotoxic Activities | [30] |
| 23. | Biphenyl-2,3-Diol | Hydroxybiphenyls | 254 | *Magnolia Garrettii* | Antifungal Activity, Fungicide and Antiseptic | [30] |
| 24. | Resveratrol | Phenolic | 445154 | *Fallopia Japonica* | Antifungal, Antitumor, Antioxidant, Antiviral, and Phytoestrogenic Agent | [31] |
| 25. | Chalcone | Flavonoid | 637760 | *Pisum Sativum* | Antifungal Activity, Antibacterial, Antimalarial, Antiprotozoal, Antitubercular and Anticancer activity | [32] |
| 26. | Psoralen | Linear Furanocoumarins | 6199 | *Psoralea Corylifolia L. (Leguminosae)* | Antifungal Activity, Intercalates With DNA, Inhibiting DNA Synthesis, Cell Division and Antibacterial activity | [33] |
| 27. | Quercetin | Flavonoid | 5280343 | *Oak Forest* | A Potent Antioxidant, Anti-Inflammatory, Antibacterial, Antiviral, Radical-Scavenging and Inhibit Biofilm Formation In Resistance Fungus | [34] |
| 28. | Hecogenin | Steroidal Saponins | 91453 | *Agave Sisalana* | Antifungal Activity, Anti-Inflammatory, Antioxidant, Hypotensive, Anti-Hyperalgesic and Anti-Nociceptive | [35] |
| 29. | Jatrorrhizine | Alkaloid | 72323 | *Mahonia*  *Aquifol* | Antifungal Activity, Anticancer Anti-Microbial, Detoxification, Bactericidal, Hypoglycemic, Hypolipidemic and Antiparasitic Properties | [36] |
| 30. | Parthenolid | Sesquiterpene Lactone | 7251185 | *Tanacetum Parthenium* | Antifungal Activity, Anti-Inflammatory and Anti-Cancer Effects | [37] |

**References:**

[9] Lee, H.-S. and Y. Kim, *Myricetin disturbs the cell wall integrity and increases the membrane permeability of Candida albicans.* 2022.

[10] EL-Hefny, M., et al., *Essential and recovery oils from Matricaria chamomilla flowers as environmentally friendly fungicides against four fungi isolated from cultural heritage objects.* Processes. **7**(11) 2019, p. 809.

[11] Lira, M.H.P.d., et al., *Antimicrobial activity of geraniol: An integrative review.* Journal of Essential Oil Research. **32**(3) 2020, p. 187-197.

[12] de Oliveira Lima, M., et al., *Investigation of the antifungal potential of linalool against clinical isolates of fluconazole resistant Trichophyton rubrum.* Journal de mycologie medicale. **27**(2) 2017, p. 195-202.

[13] Galvez-Llompart, M., et al., *Molecular Topology QSAR Strategy for Crop Protection: New Natural Fungicides with Chitin Inhibitory Activity.* ACS omega. **5**(27) 2020, p. 16358-16365.

[14] Janeczko, M., *Emodin Reduces the Activity of (1, 3)--D-glucan Synthase from and Does Not Interact with Caspofungin.* Polish Journal of Microbiology. **67**(4) 2018, p. 463-470.

[15] Pitarokili, D., et al., *Volatile metabolites from Salvia fruticosa as antifungal agents in soilborne pathogens.* Journal of agricultural and food chemistry. **51**(11) 2003, p. 3294-3301.

[16] Kusumoto, N., et al., *Antifungal properties of terpenoids in P icea abies against H eterobasidion parviporum.* Forest pathology. **44**(5) 2014, p. 353-361.

[17] Morteza-Semnani, K., et al., *Antifungal activity of the methanolic extract and alkaloids of Glaucium oxylobum.* Fitoterapia. **74**(5) 2003, p. 493-496.

[18] Yang, D., et al., *Use of caryophyllene oxide as an antifungal agent in an in vitro experimental model of onychomycosis.* Mycopathologia. **148**(2) 2000, p. 79-82.

[19] Venkatakrishnan, K., et al., *Effects of the antifungal agents on oxidative drug metabolism: clinical relevance.* Clinical pharmacokinetics. **38**2000, p. 111-180.

[20] Marchese, A., et al., *Antibacterial and antifungal activities of thymol: A brief review of the literature.* Food chemistry. **210**2016, p. 402-414.

[21] Chami, N., et al., *Antifungal treatment with carvacrol and eugenol of oral candidiasis in immunosuppressed rats.* Brazilian Journal of Infectious Diseases. **8**(3) 2004, p. 217-226.

[22] Klein, K., et al., *Identification of Cryptococcus gattii by use of L-canavanine glycine bromothymol blue medium and DNA sequencing.* Journal of clinical microbiology. **47**(11) 2009, p. 3669-3672.

[23] Schweizer, P., et al., *Effect of jasmonic acid on the interaction of barley (Hordeum vulgare L.) with the powdery mildew Erysiphe graminis f. sp. hordei.* Plant Physiology. **102**(2) 1993, p. 503-511.

[24] von Moltke, L.L., et al., *Inhibition of terfenadine metabolism in vitro by azole antifungal agents and by selective serotonin reuptake inhibitor antidepressants: relation to pharmacokinetic interactions in vivo.* Journal of clinical psychopharmacology. **16**(2) 1996, p. 104-112.

[25] Davis, S.R., *An overview of the antifungal properties of allicin and its breakdown products–the possibility of a safe and effective antifungal prophylactic.* Mycoses. **48**(2) 2005, p. 95-100.

[26] Ivanov, M., et al., *Rosmarinic acid–modes of antimicrobial and antibiofilm activities of a common plant polyphenol.* South African Journal of Botany. **146**2022, p. 521-527.

[27] Singh, S., et al., *Phytochemistry and Pharmacological Profile of Drumstick Tree “Moringa oleifera Lam”: An Overview.* Current Nutrition & Food Science. **19**(5) 2023, p. 529-548.

[28] Jothi, R., et al., *Catechol thwarts virulent dimorphism in Candida albicans and potentiates the antifungal efficacy of azoles and polyenes.* Scientific Reports. **11**(1) 2021, p. 21049.

[29] Shreaz, S., et al., *Cinnamaldehyde and its derivatives, a novel class of antifungal agents.* Fitoterapia. **112**2016, p. 116-131.

[30] Fernandez-San Millan, A., et al., *Identification of new antifungal metabolites produced by the yeast Metschnikowia pulcherrima involved in the biocontrol of postharvest plant pathogenic fungi.* Postharvest Biology and Technology. **192**2022, p. 111995.

[31] Vestergaard, M. and H. Ingmer, *Antibacterial and antifungal properties of resveratrol.* International Journal of Antimicrobial Agents. **53**(6) 2019, p. 716-723.

[32] Gupta, D. and D. Jain, *Chalcone derivatives as potential antifungal agents: Synthesis, and antifungal activity.* Journal of advanced pharmaceutical technology & research. **6**(3) 2015, p. 114.

[33] Yu, X., et al., *Design, synthesis and antifungal activity of psoralen derivatives.* Molecules. **22**(10) 2017, p. 1672.

[34] Takahama, U. and S. Hirota, *Deglucosidation of quercetin glucosides to the aglycone and formation of antifungal agents by peroxidase-dependent oxidation of quercetin on browning of onion scales.* Plant and Cell Physiology. **41**(9) 2000, p. 1021-1029.

[35] Yang, C.-R., et al., *Antifungal activity of C-27 steroidal saponins.* Antimicrobial agents and chemotherapy. **50**(5) 2006, p. 1710-1714.

[36] Volleková, A., et al., *Antifungal activity of Mahonia aquifolium extract and its major protoberberine alkaloids.* Phytotherapy Research. **17**(7) 2003, p. 834-837.

[37] Ahmed, S.M. and S.A. Abdelgaleil, *Antifungal activity of extracts and sesquiterpene lactones from Magnolia grandiflora L.(Magnoliaceae).* Int. J. Agric. Biol. **7**2005, p. 638-642.
