# Supplementary material for: Antifungal plant flavonoids identified in silico with potential to control rice blast disease caused by Magnaporthe oryzae
Source: PLoS One. 2024 Apr 5;19(4):e0301519. doi: 10.1371/journal.pone.0301519 (PMC10997076; doi:10.1371/journal.pone.0301519)
Supplement: S4 Table — (DOCX) [file pone.0301519.s010.docx]

**S4 Table:** Docking result of target protein against metabolites

|  |  |  | HDock Server |  | CB Dock |
| --- | --- | --- | --- | --- | --- |
| SI No | Protein Name (receptor) | Name of Metabolites (Ligands) | Docking Score | Confidence Score | Vina Score |
|  | ML | Rosmarinic Acid | -211.31 | 0.7732 | -9.6 |
|  |  | Myricetin | -204.42 | 0.7481 | -9.6 |
|  |  | 2-Coumaroylquinic Acid | -203.47 | 0.7445 | -8.6 |
|  |  | Quercetin | -196.15 | 0.7157 | -9.6 |
|  |  | Azoxystrobin (Ref Drug) | -176.87 | 0.6312 | -8.4 |
|  |  | Hecogenin | -175.56 | 0.6251 | -8.6 |
|  |  | Jatrorrhizine | -174.82 | 0.6216 | -8.6 |
|  |  | Emodin | -168.18 | 0.5899 | -9.4 |
|  |  | Protoemetine | -155.90 | 0.5295 | -7 |
|  |  | Resveratrol | -150.64 | 0.5032 | -8.5 |
|  |  | Glaucine | -148.79 | 0.4940 | -7.4 |
|  |  | Jasmonic Acid | -138.14 | 0.4410 | -6.4 |
|  |  | Chalcone | -137.97 | 0.4401 | -8.4 |
|  |  | Sparteine | -135.61 | 0.4285 | -8 |
|  |  | Sinapaldehyde | -135.50 | 0.4280 | -6.6 |
|  |  | Psoralen | -133.94 | 0.4204 | -7.8 |
|  |  | Parthenolide | -129.54 | 0.3991 | -7.7 |
|  |  | Serotonin | -126.17 | 0.3831 | -7.1 |
|  |  | Biphenyl-2,3-Diol | -125.19 | 0.3784 | -8 |
|  |  | 1-Methylxanthine | -122.86 | 0.3675 | -6 |
|  |  | Chamazulene | -117.36 | 0.3424 | -7.3 |
|  |  | Piperitenone Oxide | -113.88 | 0.3269 | -6.1 |
|  |  | Bornyl Acetate | -112.55 | 0.3210 | -6.3 |
|  |  | Linalool | -104.38 | 0.2865 | -5.9 |
|  |  | Carvacrol | -103.56 | 0.2832 | -6.9 |
|  |  | Thymol | -103.22 | 0.2818 | -6.5 |
|  |  | Geraniol | -100.73 | 0.2718 | -6.2 |
|  |  | Tricyclazole(Ref Drug) | -96.46 | 0.2553 | -7.4 |
|  |  | Camphor | -95.14 | 0.2503 | -5.5 |
|  |  | Canavanine | -90.69 | 0.2339 | -6.5 |
|  |  | Catechol | -90.50 | 0.2333 | -5.3 |
|  |  | Allicin | -74.28 | 0.1803 | -4.7 |
|  | PMSF | Myricetin | -143.36 | 0.4668 | -6.7 |
|  |  | Rosmarinic Acid | -142.90 | 0.4646 | -7 |
|  |  | Quercetin | -141.30 | 0.4566 | -6.6 |
|  |  | 2-Coumaroylquinic Acid | -140.56 | 0.4529 | -6.3 |
|  |  | Azoxystrobin (Ref Drug) | -137.86 | 0.4396 | -6.8 |
|  |  | Hecogenin | -136.81 | 0.4344 | -8.7 |
|  |  | Protoemetine | -131.18 | 0.4070 | -6.3 |
|  |  | Glaucine | -126.13 | 0.3829 | -6.3 |
|  |  | Jatrorrhizine | -124.15 | 0.3736 | -6.6 |
|  |  | Emodin | -122.78 | 0.3672 | -6.5 |
|  |  | Parthenolide | -104.47 | 0.2869 | -6.5 |
|  |  | Resveratrol | -109.21 | 0.3067 | -6.2 |
|  |  | Sinapaldehyde | -99.62 | 0.2674 | -5 |
|  |  | Biphenyl-2,3-Diol | -97.11 | 0.2577 | -6 |
|  |  | Chalcone | -96.60 | 0.2558 | -6.2 |
|  |  | Psoralen | -95.83 | 0.2529 | -5.1 |
|  |  | Jasmonic Acid | -91.39 | 0.2365 | -4.6 |
|  |  | Sparteine | -91.39 | 0.2365 | -5.4 |
|  |  | Chamazulene | -84.81 | 0.2135 | -5.3 |
|  |  | Bornyl Acetate | -84.35 | 0.2120 | -5.1 |
|  |  | Serotonin | -82.49 | 0.2058 | -5.2 |
|  |  | 1-Methylxanthine | -81.14 | 0.2015 | -4.7 |
|  |  | Piperitenone Oxide | -80.88 | 0.2006 | -4.5 |
|  |  | Geraniol | -75.29 | 0.1833 | -4.2 |
|  |  | Linalool | -75.25 | 0.1832 | -4.3 |
|  |  | Thymol | -75.20 | 0.1830 | -4.5 |
|  |  | Carvacrol | -74.87 | 0.1820 | -4.6 |
|  |  | Tricyclazole(Ref Drug) | -73.55 | 0.1781 | -5.3 |
|  |  | Canavanine | -71.58 | 0.1724 | -4.5 |
|  |  | Camphor | -71.17 | 0.1713 | -4.6 |
|  |  | Catechol | -63.16 | 0.1497 | -3.9 |
|  |  | Allicin | -54.38 | 0.1287 | -3.6 |
|  | CP2 | 2-Coumaroylquinic Acid | -206.22 | 0.7548 | -9 |
|  |  | Rosmarinic Acid | -196.92 | 0.7188 | -9.3 |
|  |  | Azoxystrobin (Ref Drug) | -194.55 | 0.7091 | -9.6, -8.9 |
|  |  | Hecogenin | -191.82 | 0.6977 | -9.1 |
|  |  | Myricetin | -190.29 | 0.6912 | -10.1 |
|  |  | Quercetin | -181.77 | 0.6537 | -10.6 |
|  |  | Jatrorrhizine | -180.38 | 0.6474 | -9.2 |
|  |  | Glaucine | -179.38 | 0.6428 | -9.3 |
|  |  | Emodin | -168.09 | 0.5895 | -7.5 |
|  |  | Protoemetine | -162.35 | 0.5614 | -8.3 |
|  |  | Resveratrol | -146.87 | 0.4844 | -8.1 |
|  |  | Sinapaldehyde | -139.38 | 0.4471 | -6.7 |
|  |  | Parthenolide | -135.86 | 0.4298 | -8.3 |
|  |  | Psoralen | -137.25 | 0.4366 | -7.7 |
|  |  | Chalcone | -132.10 | 0.4114 | -8 |
|  |  | Serotonin | -125.78 | 0.3812 | -6.6 |
|  |  | 1-Methylxanthine | -125.35 | 0.3792 | -6.9 |
|  |  | Jasmonic Acid | -124.88 | 0.3770 | -6.9 |
|  |  | Biphenyl-2,3-Diol | -123.42 | 0.3701 | -7.4 |
|  |  | Sparteine | -119.18 | 0.3506 | -8.1 |
|  |  | Chamazulene | -116.79 | 0.3398 | -8.8 |
|  |  | Piperitenone Oxide | -114.87 | 0.3312 | -6.4 |
|  |  | Bornyl Acetate | -114.14 | 0.3280 | -7.3 |
|  |  | Thymol | -111.46 | 0.3163 | -6.1 |
|  |  | Carvacrol | -111.21 | 0.3152 | -6.2 |
|  |  | Tricyclazole (Ref Drug) | -101.28 | 0.2740 | -7.1 |
|  |  | Camphor | -99.47 | 0.2669 | -6.1 |
|  |  | Geraniol | -96.34 | 0.2548 | -5.8 |
|  |  | Linalool | -95.98 | 0.2534 | -5.6 |
|  |  | Catechol | -94.08 | 0.2463 | -5.5 |
|  |  | Canavanine | -87.54 | 0.2228 | -6.4 |
|  |  | Allicin | -72.55 | 0.1752 | -4.9 |
|  | HPNST | Rosmarinic Acid | -198.49 | 0.7251 | -9.7 |
|  |  | Myricetin | -195.25 | 0.7120 | -9.3 |
|  |  | Hecogenin | -191.96 | 0.6983 | -9.1 |
|  |  | Quercetin | -186.41 | 0.6744 | -9.4 |
|  |  | Azoxystrobin (Ref Drug) | -176.47 | 0.6293 | -7.9 |
|  |  | 2-Coumaroylquinic Acid | -174.15 | 0.6185 | -9 |
|  |  | Jatrorrhizine | -162.38 | 0.5616 | -8 |
|  |  | Emodin | -160.47 | 0.5522 | -7.5 |
|  |  | Glaucine | -159.42 | 0.5470 | -8.2 |
|  |  | Protoemetine | -152.38 | 0.5119 | -7.6 |
|  |  | Chalcone | -139.62 | 0.4483 | -8.3 |
|  |  | Resveratrol | -135.71 | 0.4290 | -8.4 |
|  |  | Psoralen | -129.38 | 0.3983 | -7.2 |
|  |  | Sinapaldehyde | -125.97 | 0.3821 | -6.7 |
|  |  | Jasmonic Acid | -125.50 | 0.3799 | -6.7 |
|  |  | Parthenolide | -122.99 | 0.3681 | -8 |
|  |  | 1-Methylxanthine | -120.39 | 0.3561 | -6.1 |
|  |  | Biphenyl-2,3-Diol | -119.88 | 0.3538 | -7.7 |
|  |  | Sparteine | -119.58 | 0.3524 | -8.1 |
|  |  | Serotonin | -116.67 | 0.3393 | -6.7 |
|  |  | Chamazulene | -110.98 | 0.3142 | -7.9 |
|  |  | Piperitenone Oxide | -108.66 | 0.3043 | -6 |
|  |  | Bornyl Acetate | -103.15 | 0.2815 | -5.8 |
|  |  | Carvacrol | -99.95 | 0.2687 | -6.7 |
|  |  | Thymol | -97.38 | 0.2588 | -6.7 |
|  |  | Geraniol | -96.68 | 0.2561 | -5.8 |
|  |  | Tricyclazole (Ref Drug) | -95.79 | 0.2527 | -6.7 |
|  |  | Linalool | -94.30 | 0.2471 | -6.2 |
|  |  | Camphor | -88.12 | 0.2249 | -6.1 |
|  |  | Canavanine | -88.11 | 0.2248 | -5.6 |
|  |  | Catechol | -85.56 | 0.2161 | -4.7 |
|  |  | Allicin | -67.78 | 0.1619 | -4.6 |
