# Supplementary material for: Antifungal plant flavonoids identified in silico with potential to control rice blast disease caused by Magnaporthe oryzae
Source: PLoS One. 2024 Apr 5;19(4):e0301519. doi: 10.1371/journal.pone.0301519 (PMC10997076; doi:10.1371/journal.pone.0301519)
Supplement: S5 Table — (DOCX) [file pone.0301519.s011.docx]

**S5 Table:** Toxicity parameter of selected metabolites

| **Toxicity Parameters** | **Compound Name** | | | |
| --- | --- | --- | --- | --- |
|  | **Rosmarinic acid** | **Myricetin** | **2-Coumaroylquinic acid** | **Quercetin** |
| **AMES Toxicity** | No | No | No | No |
| **Max. Tolerated Dose (log mg/kg/day)** | 0.152 | 0.51 | -0.09 | 0.499 |
| **hERG I inhibitor** | No | No | No | No |
| **hERG II inhibitors** | No | No | No | No |
| **Oral Rat Acute Toxicity, LD50 (mol/kg)** | 2.811 | 2.497 | 1.737 | 2.471 |
| **Oral Rat Chronic Toxicity,LOAEL (log mg/kg_bw/day)** | 2.907 | 2.718 | 2.508 | 2.612 |
| **Hepatotoxicity** | No | No | No | No |
| **Skin Sensitisation** | No | No | No | No |
| ***T.Pyriformis* toxicity** | 0.302 | 0.286 | 0.285 | 0.288 |
| **Minnow Toxicity (log mM)** | 2.698 | 5.023 | 4.611 | 3.721 |
