# Supplementary material for: Antifungal plant flavonoids identified in silico with potential to control rice blast disease caused by Magnaporthe oryzae
Source: PLoS One. 2024 Apr 5;19(4):e0301519. doi: 10.1371/journal.pone.0301519 (PMC10997076; doi:10.1371/journal.pone.0301519)
Supplement: S6 Table — (DOCX) [file pone.0301519.s012.docx]

**S6 Table:** Bioactivity assay prediction of top metabolites

| **Name of Compounds** | **2-Coumaroylquinic acid** | **Myricetin** | **Rosmarinic Acid** | **Quercetin** |
| --- | --- | --- | --- | --- |
| **GPCR ligand** | 0.31 | -0.06 | 0.17 | -0.06 |
| **Ion channel modulator** | 0.17 | -0.18 | -0.08 | -0.19 |
| **Kinase inhibitor** | 0.02 | 0.28 | -0.18 | 0.28 |
| **Nuclear receptor ligand** | 0.80 | 0.32 | 0.57 | 0.36 |
| **Protease inhibitor** | 0.29 | -0.20 | 0.15 | -0.25 |
| **Enzyme inhibitor** | 0.65 | 0.30 | 0.24 | 0.28 |
